# An enhanced YOLOv10 architecture for high-sensitivity and high-specificity lung cancer detection

**DOI:** 10.3389/fonc.2025.1698698

**Published:** 2026-01-02

**Authors:** Liqun Li, Jing Guo, Yunfei Li, Chendong Li, Jiao Du

**Affiliations:** 1 Qingdao Municipal Hospital, University of Health and Rehabilitation Sciences, Qingdao, China; 2 Department of Research and Development, Huawei Technologies Co., Ltd, Nanjing, China

**Keywords:** lung cancer detection, CT imaging, object detection, YOLOv10, computer-aided diagnosis

## Abstract

Lung cancer detection using computed tomography (CT) imaging is a critical task for early diagnosis and improved patient outcomes. However, accurate identification of small and low-contrast pulmonary nodules remains challenging due to variations in nodule size, shape, and complex background interference. To overcome these challenges, we propose HARM-YOLO, an enhanced object detection framework based on YOLOv10, specifically designed for lung cancer detection in CT scans. Our model incorporates a multi-dimensional receptive field feature extractor (C2f-MDR), a decoupled neck architecture (DENeck), series and parallel receptive field enhancement modules (SRFEM and PRFEM), and a background attention mechanism to strengthen multi-scale feature representation and suppress irrelevant signals. Extensive experiments on the LIDC-IDRI and LUNA16 datasets demonstrate that HARM-YOLO achieves a mean average precision (mAP@0.5) of 91.3% and sensitivity of 92.7%, outperforming state-of-the-art methods including YOLOv5, ELCT-YOLO, and MSG-YOLO by significant margins. With an optimal balance of 92.7% sensitivity and 89.7% precision, our framework effectively detects true nodules while minimizing false positives, addressing key needs for computer-aided diagnosis in clinical screening. Furthermore, compared against segmentation-based approaches such as nnUNet and Swin-UNet, HARM-YOLO maintains superior performance on small nodules (≤6 mm) and real-time inference speed suitable for large-scale lung cancer screening programs. Our results highlight the potential of this YOLOv10-based object detection system as a robust and efficient tool for enhancing early lung cancer detection and supporting clinical decision-making.

## 1 Introduction

Lung cancer remains one of the leading causes of cancer-related mortality worldwide (1), accounting for more than one million deaths annually (2). Early detection is crucial for improving patient survival (3), yet small pulmonary nodules often present subtle radiological features that are difficult to distinguish from benign lesions or surrounding tissues (4). Computed tomography (CT) has become the standard imaging modality for lung cancer screening due to its high sensitivity (5); however, the manual interpretation of CT scans is time-consuming, subject to inter-observer variability, and limited in detecting subtle or low-contrast lesions (6). These challenges highlight the need for accurate and efficient computer-aided diagnostic (CAD) systems to support radiologists in early lung cancer detection (7).

Recent advances in deep learning, particularly convolutional neural networks (CNNs), have significantly improved automated medical image analysis (8). CNN-based methods have been widely applied for both classification and detection of lung nodules (9), providing robust feature extraction and improved generalization compared with traditional machine learning techniques (10). Among them, object detection models have gained increasing attention because they can simultaneously localize and classify lesions (11). YOLO (You Only Look Once) models, in particular, are known for their balance between accuracy and computational efficiency, enabling real-time detection (12). Nevertheless, existing YOLO-based approaches face persistent limitations in medical imaging tasks, such as difficulties in identifying small nodules, handling low-contrast lesions, and reducing false positives caused by background structures (13).

Addressing these challenges requires models that can capture fine-grained spatial details, integrate multi-scale contextual information, and effectively suppress irrelevant background signals. To this end, we propose HARM-YOLO, an enhanced YOLOv10-based detection framework specifically designed for lung cancer detection in CT scans. The model incorporates several key innovations: a multi-dimensional receptive field feature extractor (C2f-MDR) to capture subtle lesion patterns; a decoupled neck (DENeck) to mitigate semantic conflicts across scales; series and parallel receptive field enhancement modules (SRFEM and PRFEM) to improve multi-scale representation; and a background attention mechanism to reduce false positives. Together, these components aim to increase sensitivity to small and low-contrast nodules while maintaining the efficiency required for real-time screening.

This work addresses a critical gap in automated lung cancer screening by simultaneously tackling three fundamental challenges that have limited clinical deployment of existing detection systems: (1) the persistent difficulty in reliably identifying small nodules (≤6mm) that represent the earliest detectable stage of malignancy, (2) the high false positive rates caused by anatomical mimics such as vessels and pleural interfaces, and (3) the lack of interpretable decision mechanisms that would enable radiologist trust and adoption in clinical workflows.

The primary objective of this study is to develop a detection framework that achieves clinically meaningful sensitivity improvements for small and low-contrast nodules while maintaining real-time inference capability suitable for large-scale screening programs. Unlike prior approaches that prioritize either speed or accuracy, our framework is designed to satisfy both requirements concurrently through principled architectural innovations rather than model scaling alone. Specifically, we target three quantifiable goals: (1) achieving >90% sensitivity for nodules ≤6mm on benchmark datasets, (2) reducing false positive rates by at least 15% compared to YOLOv10 baseline through targeted background suppression, and (3) maintaining inference speed suitable for processing >100 CT volumes per hour on standard clinical hardware.

To accomplish these objectives, we introduce HARM-YOLO, an enhanced detection framework built upon YOLOv10 with four synergistic architectural innovations. The C2f-MDR module expands receptive fields across multiple dimensions to capture subtle morphological patterns characteristic of early-stage lesions. The DENeck architecture decouples semantic pathways to prevent feature conflicts across scales, addressing a fundamental limitation in conventional feature pyramid networks. Series and parallel receptive field enhancement modules (SRFEM and PRFEM) provide complementary multi-scale representations, enabling simultaneous encoding of fine-grained texture and global context. Finally, a background attention mechanism explicitly models the distinction between nodule features and anatomical structures, substantially reducing false positives without sacrificing sensitivity.

The contributions of this study extend beyond incremental performance gains to provide fundamental insights into medical object detection. *First*, we demonstrate that receptive field engineering, when properly designed for medical imaging characteristics, yields more substantial improvements than generic architectural scaling—our ablation studies show that SRFEM and PRFEM together contribute 4.8% absolute gain in mAP@0.5 while adding minimal computational overhead. *Second*, we establish through rigorous cross-dataset validation (LIDC-IDRI ↔ LUNA16) that decoupled semantic processing significantly enhances generalization across different imaging protocols and annotation standards, a critical requirement for real-world deployment. *Third*, we provide the first comprehensive interpretability analysis of YOLO-based lung nodule detection using both Grad-CAM and Score-CAM, revealing that our attention mechanisms reliably focus on clinically relevant features rather than spurious correlations, thereby addressing a key barrier to clinical acceptance. *Fourth*, we introduce a statistically rigorous evaluation framework including FROC analysis at clinically relevant operating points (1–8 FPs/scan), demonstrating that HARM-YOLO achieves 89.5% sensitivity at 1 FP/scan—a 5.4% absolute improvement over the strongest baseline and approaching the performance requirements for clinical screening programs.

The clinical significance of this work lies not merely in achieving state-of-the-art benchmark performance, but in demonstrating that principled architectural design can overcome fundamental limitations that have prevented widespread adoption of automated detection systems. By achieving high sensitivity for small nodules while maintaining specificity and real-time speed, HARM-YOLO represents a meaningful step toward scalable computer-aided lung cancer screening that can augment radiologist performance in early detection, potentially improving patient outcomes through earlier intervention.

In summary, this work advances the field of computer-aided lung cancer detection by introducing a robust, interpretable, and computationally efficient framework. The proposed HARM-YOLO not only addresses key limitations of existing YOLO-based methods but also provides a promising solution for large-scale lung cancer screening, with the potential to support earlier diagnosis and improved patient outcomes.

## 2 Related work

### 2.1 Traditional machine learning approaches

Before the emergence of deep learning, lung cancer detection in CT imaging largely relied on traditional machine frameworks that combined radiological imaging with handcrafted feature engineering (14). In these approaches, researchers extracted features such as intensity histograms, edge and gradient descriptors, gray-level co-occurrence matrices (GLCM), local binary patterns (LBP), and shape descriptors to characterize pulmonary nodules (15). These handcrafted features were then input into classical classifiers including support vector machines (SVM), decision trees, k-nearest neighbors (k-NN), and random forests. While these methods were computationally efficient and interpretable, their performance was strongly dependent on the quality of feature design, which required expert knowledge and often lacked robustness when applied to diverse clinical datasets (16, 17).

A key advantage of traditional approaches was their interpretability, as clinicians could relate extracted features to clinically meaningful descriptors such as nodule texture or spiculation. However, these methods struggled with scalability and generalization. Handcrafted features tended to be highly sensitive to imaging noise, acquisition protocol differences, and subtle inter-patient variability in nodule appearance. Furthermore, these methods were limited in their ability to detect small nodules or nodules with low contrast against surrounding lung tissue. They typically required pre-segmented regions of interest (ROIs), meaning that accurate detection was contingent on prior nodule localization by radiologists. As a result, such systems often suffered from high false-negative rates and poor clinical applicability.

Despite their limitations, these early methods established important foundations in computer-aided detection (CAD) systems. They highlighted the need for automated systems that can generalize across heterogeneous imaging data and motivated the transition towards feature learning approaches, eventually leading to the adoption of deep neural networks.

### 2.2 CNN-based classification models

The introduction of convolutional neural networks (CNNs) marked a significant paradigm shift in medical image analysis by eliminating the need for manual feature engineering (18). Early studies adopted popular architectures such as VGGNet (19) and ResNet (20), applying them to lung nodule classification tasks. By automatically learning hierarchical features from raw CT images, these models substantially outperformed handcrafted feature-based methods in terms of accuracy and generalization. They demonstrated that low-level patterns such as edges and textures, as well as high-level abstract features, could be effectively captured in an end-to-end learning framework (21, 22).

Subsequent work extended CNNs to three-dimensional architectures, leveraging the volumetric nature of CT scans. 3D CNNs provided richer contextual information by analyzing entire nodule volumes rather than isolated 2D slices, leading to improved malignancy prediction and nodule characterization. These models achieved promising results in public benchmarks and clinical trials, showing their potential for computer-aided diagnosis (23, 24).

However, CNN-based classification models faced inherent limitations. Their primary focus was binary or multi-class classification of nodules (e.g., benign *vs*. malignant), rather than joint localization and classification. As such, they did not provide bounding boxes or segmentation masks to indicate the precise position and extent of detected nodules, limiting their usability in clinical workflows where accurate localization is critical (25). Moreover, these models often required manually cropped nodule patches as input, which again depended on prior annotation or preprocessing. Another major challenge was their reduced sensitivity to small nodules and low-contrast lesions, which remain the most clinically significant cases for early detection.

In summary, CNN-based classification approaches demonstrated the feasibility and advantages of deep learning in lung cancer detection but did not fully address the clinical requirements of automated, real-time, and fine-grained detection. These shortcomings paved the way for the adoption of object detection frameworks, which offer a more comprehensive solution by simultaneously localizing and classifying pulmonary nodules.

### 2.3 Object detection frameworks in medical imaging

To overcome the limitations of pure classification approaches, object detection frameworks have been increasingly adopted in lung cancer research (26). These methods are capable of simultaneously localizing and classifying nodules, thus aligning more closely with the clinical workflow. Among the earliest deep learning-based detectors, two-stage frameworks such as Faster R-CNN gained popularity due to their strong detection accuracy. Faster R-CNN employs a region proposal network (RPN) to generate candidate bounding boxes, followed by classification and refinement stages. Its ability to provide precise localization made it effective for detecting large and medium-sized nodules in CT images (27, 28). However, the reliance on region proposals and multi-stage processing resulted in high computational cost and slow inference, which limited its suitability for real-time screening (29, 30).

Single-stage detectors, such as RetinaNet, attempted to bridge the gap between accuracy and speed by eliminating the proposal stage and introducing focal loss to address class imbalance. RetinaNet demonstrated improved performance on small lesion detection compared with earlier CNN classifiers. Nevertheless, its computational complexity remained relatively high, and its performance on extremely small nodules was still unsatisfactory (31, 32).

The YOLO (You Only Look Once) series of models introduced a significant breakthrough by reframing object detection as a single regression problem. YOLOv3 and YOLOv4 were among the first variants applied to medical imaging tasks, achieving real-time detection while maintaining competitive accuracy. In the context of lung cancer detection, YOLO-based models offered notable improvements in processing speed, making them promising candidates for large-scale screening. YOLOv5 further advanced feature extraction and multi-scale fusion, delivering higher accuracy and flexibility across different hardware platforms. Despite these successes, existing YOLO models continued to face challenges when dealing with small nodules and low-contrast lesions, often resulting in false negatives. In addition, their limited mechanisms for suppressing background interference hindered robustness in complex CT scans, where anatomical structures such as blood vessels could mimic nodules (33, 34).

Overall, object detection frameworks brought detection closer to clinical applicability by addressing localization and classification simultaneously. However, balancing speed, accuracy, and sensitivity to small nodules remained an open challenge, motivating further architectural innovation.

### 2.4 Transformer-based methods

In parallel with CNN-based detection models, transformer architectures have recently gained attention in medical image analysis. Transformers, originally introduced in natural language processing, have demonstrated strong capabilities in modeling long-range dependencies and global contextual information. Their adaptation to vision tasks, such as the Vision Transformer (ViT) and Swin Transformer, has shown competitive performance on natural image classification and detection benchmarks.

In the medical domain, transformer-based models such as TransUNet and Swin-UNet have been applied to tasks including lung nodule segmentation and classification. By leveraging self-attention mechanisms, these models effectively capture global structural relationships, which is particularly beneficial in CT scans where lesions may exhibit diffuse or irregular patterns. For lung cancer detection, transformer-based frameworks have shown improved sensitivity to complex background structures and enhanced robustness against imaging variability.

Nevertheless, transformer architectures face several limitations when applied to medical object detection. Their computational demands are significantly higher than CNN-based methods, requiring extensive GPU resources and large training datasets. This constraint is particularly challenging in medical imaging, where labeled data is limited and costly to obtain. Moreover, transformer-based models generally provide stronger performance in segmentation tasks rather than real-time detection, as their inference speed is insufficient for clinical screening scenarios. As a result, while transformers have contributed new perspectives and techniques, they have not yet supplanted CNN- and YOLO-based approaches for time-sensitive lung cancer detection tasks.

### 2.5 Motivation for HARM-YOLO

The progression from handcrafted feature-based models to CNN classifiers, object detection frameworks, and more recently, transformer-based methods has substantially advanced automated lung cancer detection. Each class of approaches has addressed specific challenges, yet none has fully resolved the fundamental requirements of early and accurate lung nodule detection in CT imaging. Traditional machine learning approaches provided interpretability but were limited by their dependence on handcrafted features and poor generalization. CNN-based classification models improved feature representation but lacked the ability to simultaneously localize and classify nodules, which is critical in clinical workflows. Object detection frameworks such as Faster R-CNN, RetinaNet, and YOLO variants brought localization into focus, offering real-time detection capabilities and higher clinical relevance. However, these models still struggled with small and low-contrast nodules and were prone to false positives in complex anatomical contexts. Transformer-based approaches demonstrated potential in capturing global dependencies and structural patterns, but their computational demands and slower inference limited their applicability to large-scale, real-time screening tasks.

Motivated by these gaps, HARM-YOLO is proposed as an enhanced detection framework tailored to the specific challenges of lung cancer CT imaging. Built upon YOLOv10, the model introduces architectural innovations that target the weaknesses of existing methods. The C2f-MDR module expands receptive fields in multiple dimensions to capture subtle and small-scale lesion patterns that earlier YOLO models often missed. The DENeck module decouples feature fusion to mitigate semantic conflicts across scales, enhancing robustness in detecting nodules of varying sizes. The SRFEM and PRFEM modules improve multi-scale representation by combining serial and parallel receptive field enhancements, allowing the model to balance global context with fine-grained details. Finally, a background attention mechanism is incorporated to suppress irrelevant anatomical structures and reduce false positives, a persistent problem in CT-based detection.

By integrating these targeted improvements, HARM-YOLO bridges the gap between high detection sensitivity, computational efficiency, and clinical practicality. Unlike existing approaches, it explicitly addresses the two most critical challenges of lung cancer detection: reliable recognition of small and low-contrast nodules and robust performance in the presence of background interference. This motivates its development as a clinically oriented framework that not only achieves superior accuracy but also maintains real-time inference capability, making it highly relevant for deployment in lung cancer screening programs.

## 3 Method

The proposed HARM-YOLO framework is built upon the YOLOv10 architecture (35), which represents the state-of-the-art in real-time object detection through its NMS-free training strategy and efficient dual-head design. YOLOv10 introduces several key innovations over previous YOLO variants: (1) a streamlined backbone with CSPNet blocks for efficient feature extraction, (2) a Path Aggregation Network (PANet) for multi-scale feature fusion, (3) decoupled detection heads separating classification and localization tasks, and (4) consistent dual assignment strategy eliminating the need for Non-Maximum Suppression during inference. While these design choices enable efficient detection on natural images, lung nodule detection in CT scans presents distinct challenges—subtle low-contrast lesions, anatomical structures mimicking nodules, and extreme scale variations—that require targeted architectural modifications.

**Figure 1:**
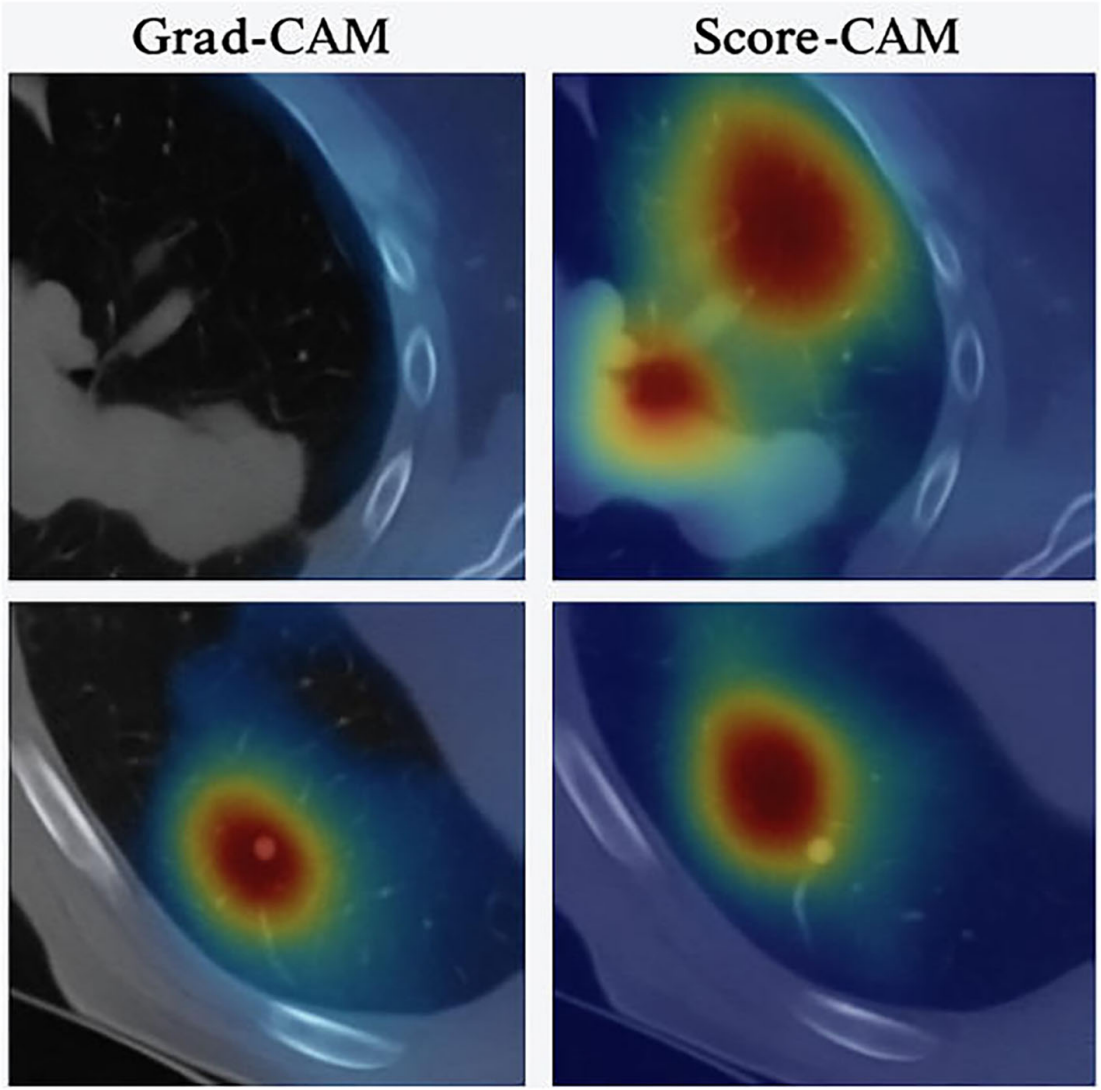
The model diagram of HARM-YOLO.

Our modifications to YOLOv10 address three fundamental limitations when applied to medical imaging. *First*, the standard CSPNet blocks in YOLOv10’s backbone employ fixed receptive fields that are insufficient for capturing the subtle textural patterns characteristic of early-stage nodules, particularly those ≤6mm. We address this by replacing key CSPNet blocks with our C2f-MDR modules, which integrate multi-dimensional dilated convolutions to simultaneously encode fine-grained details and broader anatomical context. *Second*, YOLOv10’s PANet architecture, while effective for natural images, introduces semantic conflicts when fusing features across scales in medical images where nodules exhibit highly variable appearance. We mitigate this through our DENeck module, which decouples high- and low-semantic pathways before fusion, reducing feature interference. *Third*, YOLOv10 lacks explicit mechanisms to suppress background responses from vessels, airways, and pleural interfaces that frequently cause false positives in lung CT. We augment the detection head with background attention modules and integrate SRFEM/PRFEM blocks into the neck to enhance discriminative capacity. **Figure 1** illustrates the complete HARM-YOLO architecture.

### 3.1 Function description

The HARM-YOLO framework integrates several specialized modules, each designed to address a particular aspect of the detection process. Rather than functioning in isolation, these components interact within the overall architecture to enhance both sensitivity and specificity. In what follows, the role of each module is outlined in detail, and the corresponding mathematical formulations are provided to clarify their operational principles.

#### 3.1.1 Feature extraction – C2f-MDR module

The C2f-MDR (Channel-to-Feature Multi-Dimensional Receptive Field) module constitutes a core component of the HARM-YOLO framework. Its primary function is to enhance feature extraction by introducing multi-scale receptive fields, which proves particularly beneficial for processing lung CT images where tumors exhibit diverse sizes and irregular morphologies. Rather than relying exclusively on conventional convolutional layers, this module integrates channel-to-feature interactions with receptive field expansion, thereby improving sensitivity to subtle structures that may otherwise remain undetected.

##### 3.1.1.1 Receptive field enhancement

In conventional convolutional networks, the receptive field defines the region of the input image that influences a neuron’s activation in the output feature map. While small receptive fields effectively capture local details, they often fail to encode long-range dependencies. To address this limitation, the C2f-MDR module incorporates dilated convolutions with varying dilation rates, enabling simultaneous encoding of both local and global context.

Formally, the receptive field (RF) of a convolution with kernel size *k* and dilation rate *d* is expressed as:

(1) RF=(k−1)·d+1

By employing multiple dilation rates (e.g., *d* = 1, 2, 4), the model captures hierarchical representations ranging from fine-grained textures to broader structural patterns. The overall receptive field expansion in this module is given by:

(2) $${\text{RF}}_{d_{1},d_{2},\ldots ,d_{n}}=\overset{n}{\underset{i=1}{\cup }}\text{RF}\left(k,d_{i}\right)$$

where *n* denotes the number of dilated convolutional operations. This formulation demonstrates how the module aggregates information across multiple scales to enhance tumor localization.

##### 3.1.1.2 Channel-to-feature connection

Beyond receptive field enlargement, the C2f-MDR module introduces a *channel-to-feature interaction mechanism* to improve feature fusion. This mechanism is realized through 1 × 1 convolutions, which reduce inter-channel redundancy while enhancing spatial coherence. The transformation is defined as:

(3) $$F_{\text{out}}=W\cdot F_{\text{in}}+b$$

where $F_{\text{in}}$ represents the input feature map, W and b denote the learnable weights and bias, respectively, and $F_{\text{out}}$ represents the output feature representation.

By combining receptive field expansion with efficient channel-to-feature connections, the C2f-MDR module significantly enhances the model’s capacity to capture lesions across multiple scales, thereby improving both detection accuracy and robustness in complex CT imaging scenarios.

##### 3.1.1.3 Multi-dimensional feature aggregation

To further refine feature extraction, the C2f-MDR module employs a multi-dimensional aggregation technique that combines features from different network layers. This approach enhances both the depth and breadth of feature extraction by capturing diverse spatial patterns and textures across multiple levels. The aggregation is mathematically represented as:

(4) $$F_{\text{aggregated}}=\sum_{l=1}^{L}W_{l}\cdot F_{l}$$

where $F_{l}$ represents the feature maps from layer l, $W_{l}$ denotes the learned weight for each feature map, and L is the total number of layers considered for aggregation.

This multi-dimensional feature aggregation enables the model to learn hierarchical representations of lung tissue, making it more robust to variations in tumor size, shape, and contrast.

##### 3.1.1.4 Combined feature representation

After applying dilated convolutions, channel-to-feature connections, and multi-dimensional aggregation, the final output feature map is passed through a ReLU (Rectified Linear Unit) activation function, which introduces non-linearity and enables the model to learn complex relationships. The output is represented as:

(5) $$F_{\text{final}}=\text{ReLU}\left(F_{\text{aggregated}}\right)$$

This step refines the feature maps and ensures that the model captures only the most relevant information for tumor detection.

##### 3.1.1.5 Final output

The feature representations produced by the C2f-MDR module are subsequently propagated through the downstream layers of HARM-YOLO for high-level tasks such as classification and bounding box regression. By enriching the feature maps with multi-scale contextual information, this module proves particularly valuable for identifying subtle or small lesions that conventional convolutional layers tend to overlook.

More broadly, the contribution of C2f-MDR lies in its integrated use of dilated convolutions, channel-to-feature interactions, and multi-dimensional receptive field aggregation. This design equips the network with enhanced capability to encode both fine-grained details and global structures in lung CT images. Consequently, the model achieves improved robustness in detecting low-contrast tumors and irregular morphologies. The accompanying mathematical formulations clarify how each operation contributes to this process, underscoring the module’s role as a critical component for advancing lung cancer detection accuracy.

#### 3.1.2 Decoupled neck

The Decoupled Neck (DENeck) module constitutes a critical component of the HARM-YOLO model, designed to enhance feature fusion by addressing the inherent challenges posed by the multi-scale nature of lung cancer detection. In traditional YOLO models, feature fusion typically combines features from different layers through simple concatenation or summation. However, these methods often overlook the semantic differences between features at different scales, leading to suboptimal performance, particularly when detecting small or low-contrast tumors in complex CT images. The DENeck module decouples the feature fusion process into distinct high-semantic and low-semantic branches, reducing semantic conflicts and enhancing the model’s capability to detect lesions at multiple scales.

##### 3.1.2.1 Feature decoupling and semantic separation

To address semantic conflict, the DENeck module separates high-semantic and low-semantic features into two distinct branches, each dedicated to capturing different types of information. High-semantic features typically originate from deeper network layers, capturing global context and abstract patterns, while low-semantic features derive from shallower layers, capturing finer, localized details. This decoupling enables the model to extract specific information types from different parts of the feature space without interference from irrelevant details.

Mathematically, the feature decoupling process is represented as:

(6) $${\text{F}}_{\text{high}}={\text{W}}_{\text{h}}\cdot {\text{F}}_{\text{high}-\text{semantic}}+{\text{b}}_{\text{h}}$$

(7) $${\text{F}}_{\text{low}}={\text{W}}_{\text{l}}\cdot {\text{F}}_{\text{low}-\text{semantic}}+{\text{b}}_{\text{l}}$$

where ${\text{F}}_{\text{high}}$ and ${\text{F}}_{\text{low}}$ represent the high-semantic and low-semantic features, respectively, ${\text{W}}_{\text{h}}$ and ${\text{W}}_{\text{l}}$ denote the learned weight matrices for the corresponding branches, ${\text{b}}_{\text{h}}$ and ${\text{b}}_{\text{l}}$ are the bias terms, and ${\text{F}}_{\text{high}-\text{semantic}}$ and ${\text{F}}_{\text{low}-\text{semantic}}$ represent the feature maps from deeper and shallower network layers, respectively.

##### 3.1.2.2 Feature fusion via decoupled branches

Once the features are decoupled, they undergo independent processing in separate branches to preserve their unique characteristics. The high-semantic features are processed through a series of operations aimed at refining global contextual information, while the low-semantic features are processed with operations that emphasize local details. The decoupled branches are then fused, but this fusion is done in a way that prevents semantic overlap, ensuring that each type of information contributes optimally to the final output.

The fusion process can be expressed mathematically as:

(8) $${\text{F}}_{\text{final}}=\text{Fusion}\left({\text{F}}_{\text{high}},{\text{F}}_{\text{low}}\right)$$

Where the fusion operation can be a learned weighted sum or concatenation, depending on the task at hand. The goal of the fusion process is to combine the global context provided by the high-semantic features with the detailed local information from the low-semantic features:

(9) $$F_{\text{final}}=\alpha \cdot F_{\text{high}}+\left(1-\alpha \right)\cdot F_{low}$$

Where *α* is a learned weight that determines the balance between the high and low-semantic features. The fusion operation ensures that both types of features are optimally integrated for accurate tumor detection.

##### 3.1.2.3 Scale-invariant detection via fusion

The decoupling and subsequent fusion of features improve the model’s ability to handle multi-scale tumor detection, which is crucial in lung cancer diagnosis, as tumors can vary significantly in size and contrast. The high-semantic features focus on providing global context, enabling the model to understand the broader relationships between objects in the image, while the low-semantic features focus on localized regions, making it easier to detect small or subtle lesions.

To further improve scale-invariant detection, the DENeck module introduces a multi-scale fusion mechanism that ensures both large and small tumors are effectively detected. This is done by applying scale-specific kernels to the fused feature maps, which allows the network to focus on different scales without losing essential information. The fusion of multi-scale features can be represented as:

(10) $$F_{multi-scale}=\sum_{i=1}^{n}W_{i}\cdot {\text{Scale}}_{i}\left(F_{final}\right)$$

Where $F_{multi-scale}$ is the final multi-scale feature map. $W_{i}$ is the learned weight for each scale. ${\text{Scale}}_{i}\left(F_{final}\right)$ represents the feature map after applying scale-specific operations (e.g., resizing or dilated convolutions).

By applying this multi-scale fusion mechanism, the DENeck module ensures that the model can adapt to the varying sizes of tumors and detect them more accurately, even when they are small or located in areas of the lung with poor contrast.

##### 3.1.2.4 Final output layer

After decoupling and multi-scale feature fusion, the final output features are passed through additional layers of convolution or fully connected layers, followed by a softmax or sigmoid activation function, depending on the classification task (e.g., tumor classification or bounding box regression). The final output can be represented as:

(11) $$F_{output}=\text{Activation}\left(F_{multi-scale}\right)$$

Where $F_{output}$ is the final prediction, which could be a probability map for tumor classification or the coordinates of bounding boxes for localization.

##### 3.1.2.5 Summary of the DENeck module

The DENeck module significantly improves the performance of the HARM-YOLO model by decoupling the high- and low-semantic features, reducing semantic conflicts, and enhancing multi-scale feature fusion. This design enables the model to effectively handle lung cancer detection across varying tumor sizes and complex image backgrounds, making it particularly suitable for clinical applications. The mathematical formulations presented here provide a clear understanding of how the DENeck module works to refine and fuse feature maps, ultimately improving the model’s robustness and accuracy.

#### 3.1.3 Receptive field enhancement - SRFEM and PRFEM

The SRFEM (Series Receptive Field Enhancement Module) and PRFEM (Parallel Receptive Field Enhancement Module) are designed to enlarge the network’s receptive field, enabling the capture of broader spatial context while retaining fine-grained tumor details. This capability is essential in lung cancer detection, where nodules exhibit substantial variability in size and morphology, and small lesions in particular remain difficult to identify. By extending the receptive field in complementary ways, the two modules allow the network to integrate information across larger anatomical regions while preserving local texture cues, thereby improving the accuracy of tumor localization and classification.

The SRFEM achieves receptive field expansion through a sequential application of dilated convolutions with different dilation rates. Unlike standard convolution, dilated kernels enlarge the effective field of view without adding significant computational overhead. In this design, dilation rates of 1, 3, and 5 are applied in series, enabling the network to encode hierarchical features at progressively larger scales. This sequential strategy allows the model to combine fine structural details with global contextual information. The computational process of SRFEM is formulated as (as shown in Equation 12):

(12) $${\text{y}}_{\text{SRFEM}}=\text{SiLU}\left(\text{x}+{\text{Conv}}_{3}^{5}\left({\text{Conv}}_{3}^{3}\left({\text{Conv}}_{3}^{1}\left(\text{x}\right)\right)\right)\right)$$

**Figure 2:**
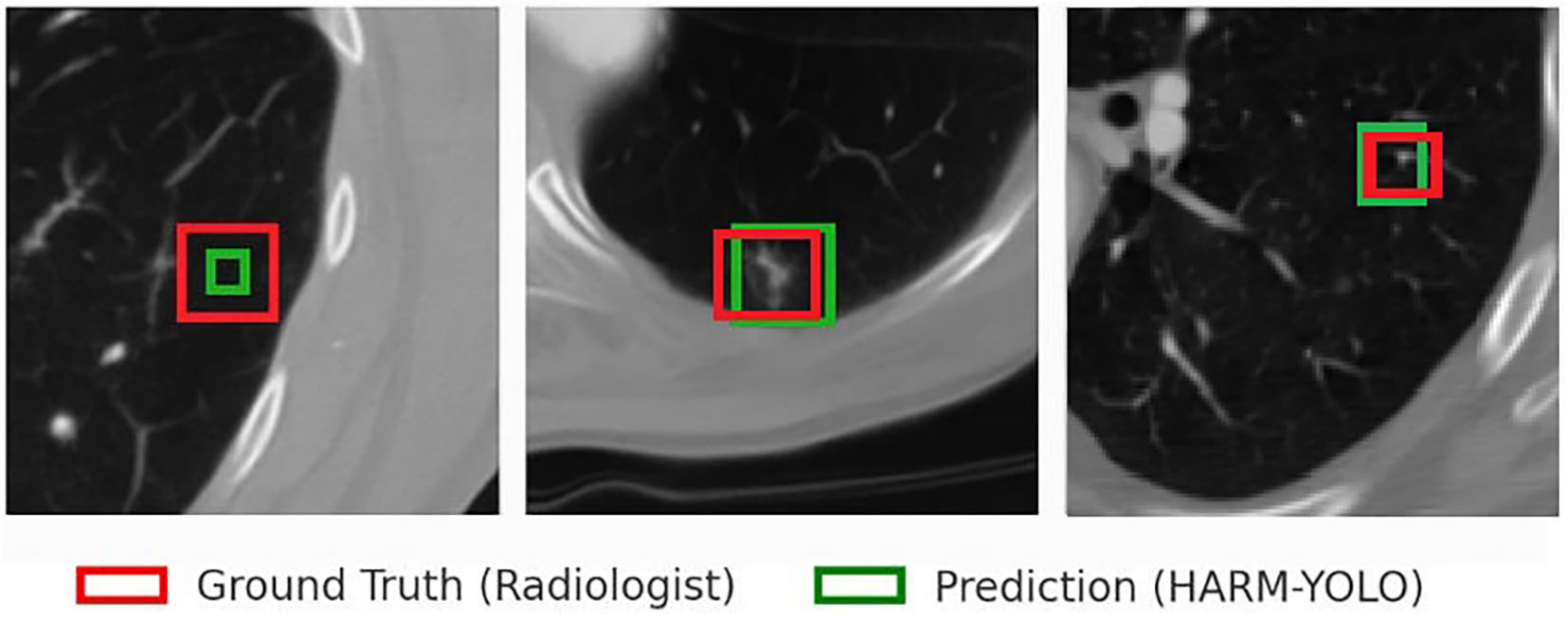
The SRFEM structure.

Where x denotes the input feature map and the superscript indicates the dilation rate of each convolutional operation. A schematic representation of the module is provided in **Figure 2**.

Through this sequential strategy, the SRFEM module extends the receptive field across multiple scales, enabling the network to combine broad contextual cues with localized structural information. Such integration is particularly advantageous for identifying small nodules whose weak contrast or irregular boundaries often complicate early detection.

In contrast to the sequential design of SRFEM, the PRFEM (Parallel Receptive Field Enhancement Module) adopts a parallel architecture. Multiple convolutional branches, each configured with a different dilation rate, operate simultaneously, and their outputs are concatenated to generate a unified feature map. This parallelization enables the module to capture features at several spatial resolutions within a single layer. The computation can be expressed as (as shown in Equation 13):

(13) $${\text{y}}_{\text{PRFEM}}=\text{Concat}\left({\text{Conv}}_{3}^{1}\left(\text{x}\right),{\text{Conv}}_{3}^{3}\left(\text{x}\right),{\text{Conv}}_{3}^{5}\left(\text{x}\right)\right)$$

**Figure 3:**
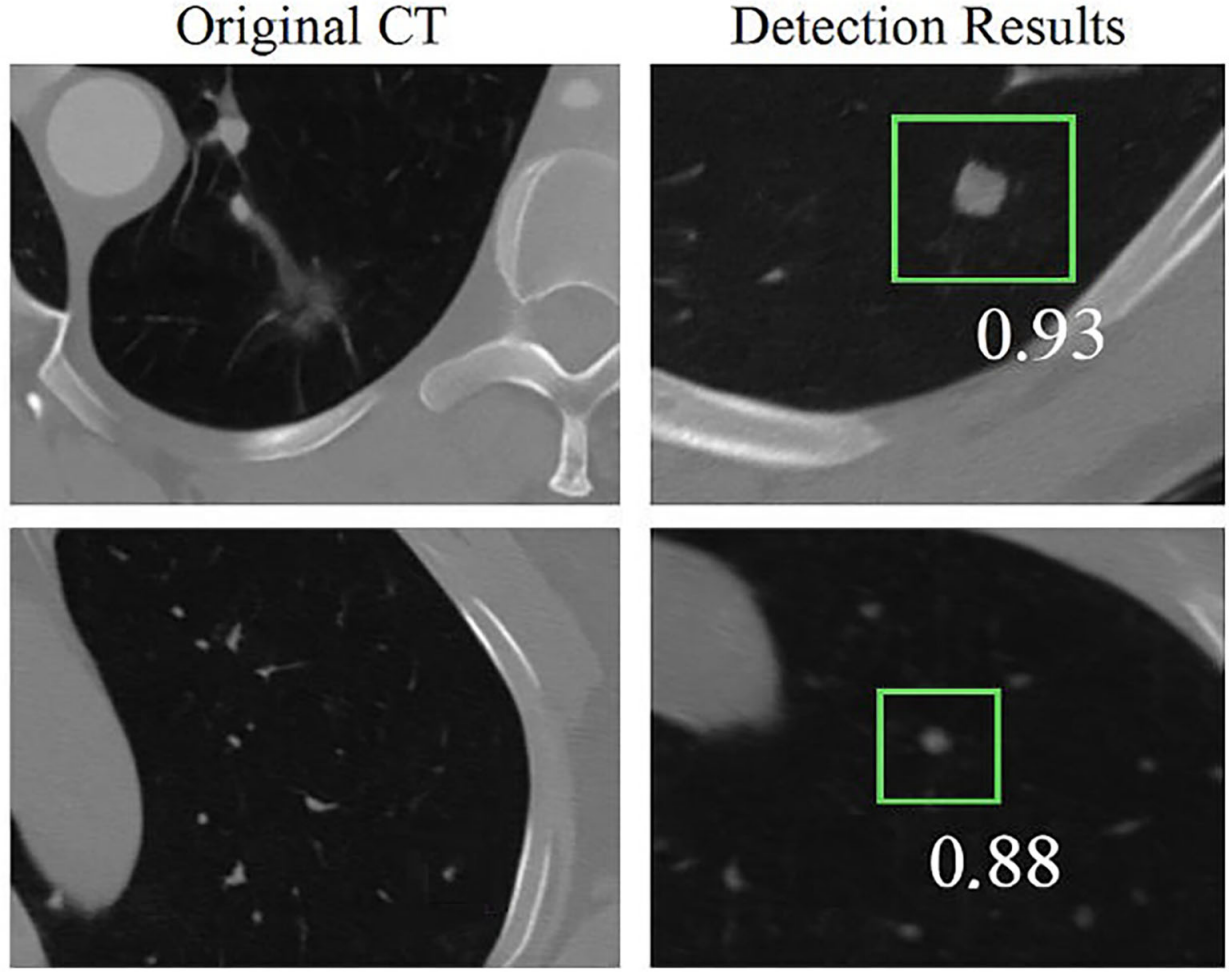
The PRFEM structure.

Where x denotes the input feature map. The architecture of PRFEM is depicted in **Figure 3**.

By aggregating features from multiple receptive fields in parallel, PRFEM improves the model’s ability to detect nodules of varying sizes and morphologies. This design is particularly effective in cases where tumors differ substantially in scale, allowing the network to maintain sensitivity to small lesions while accurately capturing the structure of larger ones.

The joint design of SRFEM and PRFEM equips HARM-YOLO with a comprehensive multi-scale feature extraction capability. By expanding receptive fields in both sequential and parallel manners, the model achieves a balance between global contextual encoding and preservation of local detail. This synergy improves both sensitivity and specificity, a property of particular importance for lung cancer screening where small nodules frequently exhibit low contrast against surrounding tissue.

Through this enhanced multi-scale representation, the two modules significantly improve detection accuracy by lowering false negatives and strengthening the discrimination between malignant nodules and normal structures. By incorporating these mechanisms, HARM-YOLO demonstrates robust performance in detecting lung tumors across different sizes and stages, thereby providing a reliable basis for computer-aided diagnosis in clinical practice.

### 3.2 Multi-level Detection Head

The Multi-level Detection Head is an essential component of the HARM-YOLO model, designed to improve the model’s detection performance by incorporating multiple levels of feature aggregation and refined output prediction. Unlike traditional models that rely on a single-level feature extraction, the Multi-level Detection Head extracts information from several intermediate layers of the network to ensure the detection of tumors across a range of scales. This approach facilitates the detection of small, low-contrast tumors, which are often missed by conventional methods. The design of the multi-level detection head integrates the information at different levels and outputs a refined prediction that improves both the accuracy and robustness of the model.

#### 3.2.1 Feature aggregation from multiple levels

The multi-level detection head operates by aggregating features from different levels of the network. These levels correspond to different depths in the feature pyramid, where higher layers capture global context, and lower layers capture fine-grained local details. The key idea is to combine these features in a way that enhances both spatial and semantic representation, facilitating the detection of tumors across varying sizes.

Let $F_{l}$ represent the feature map at level l, where l∈[1,L] indicates different layers. The aggregated feature map $F_{agg}$ at the detection head is given by (as shown in Equation 14):

(14) $$F_{agg}=\sum_{l=1}^{L}W_{l}\cdot F_{l}+b_{l}$$

Where $W_{l}$ is the learned weight matrix for the feature map $F_{l}$ at layer l, $b_{l}$ is the bias term for each layer, and L is the total number of levels considered for feature aggregation.

The aggregation process combines high-semantic features from deeper layers with low-semantic features from shallow layers, improving the overall feature representation at the detection head.

#### 3.2.2 Hierarchical multi-scale feature fusion

to handle multi-scale tumor detection, the Multi-level Detection Head applies hierarchical feature fusion. This process leverages features at multiple levels to detect both large and small tumors. By incorporating spatial pyramids and dilated convolutions, the model ensures it can handle objects of various scales and resolutions.

The fusion process combines multiple transformed feature maps using scale-specific operations, and the output is defined as (as shown in Equation 15):

(15) $$F_{multi-scale}=\sum_{i=1}^{N}W_{i}\cdot F_{i}$$

Where $F_{multi-scale}$ is the resulting fused feature map, $W_{i}$ denotes the learned weight at scale i, and $F_{i}$ represents the transformed feature map at that scale, obtained through operations such as dilated convolution with dilation rate $d_{i}$ or spatial pyramid pooling. N is the total number of scales used in the fusion.

This hierarchical fusion allows the model to integrate both fine-grained and global contextual information, improving the detection of tumors across a wide range of sizes and contrasts.

#### 3.2.3 Detection prediction via regression and classification

The core task of the multi-level detection head is to predict the bounding boxes and classify the objects within the detected regions. To achieve this, the aggregated and fused feature map $F_{multi-scale}$ is passed through a series of fully connected (FC) layers or convolutions to predict the final output. These predictions consist of two parts:

1. Bounding Box Regression: The model predicts the coordinates of the bounding boxes that enclose the detected tumors.
2. Class Prediction: The model classifies the detected tumor as either benign or malignant, or identifies the type of tumor (e.g., lung adenocarcinoma, squamous cell carcinoma).

The output can be mathematically expressed as (as shown in Equations 16, 17):

(16) $$B=\sigma \left(W_{b}\cdot F_{multi-scale}+b_{b}\right)$$

(17) $$C=\text{Softmax}\left(W_{c}\cdot F_{multi-scale}+b_{c}\right)$$

Where: B represents the bounding box predictions (coordinates of the tumor), $W_{b}$ is the learned weight matrix for the bounding box prediction, $b_{b}$ is the bias term for the bounding box prediction, C represents the classification probabilities (tumor type prediction), $W_{c}$ is the learned weight matrix for the classification prediction, $b_{c}$ is the bias term for the classification prediction.

The sigmoid activation function is used for bounding box regression, and the softmax activation is applied to the class prediction to obtain a probability distribution across different tumor types.

#### 3.2.4 Loss function for multi-level detection

The model is trained using a combined loss function that optimizes both the bounding box regression and the classification tasks. The total loss ℒ is a weighted sum of the localization loss ${ℒ}_{loc}$ and the classification loss ${ℒ}_{cls}$ (as shown in Equation 18):

(18) $$ℒ=\lambda_{loc}\cdot {ℒ}_{loc}+\lambda_{cls}\cdot {ℒ}_{cls}$$

Where ${ℒ}_{loc}=\sum_{i=1}^{N}∥B_{i}-{\hat{B}}_{i}{∥}^{2}$ is the bounding box regression loss, calculated as the squared error between the predicted and ground truth bounding boxes, ${ℒ}_{cls}=-\sum_{i=1}^{N}{\hat{C}}_{i}i\text{log}\left(C_{i}\right)$ is the classification loss, computed as the cross-entropy loss between the predicted class probabilities $C_{i}$ and the ground truth nodule labels ${\hat{C}}_{i}$. The weights $\lambda_{loc}$ and $\lambda_{cls}$ balance the contributions of localization and classification.

In this design, the ground truth annotations provided by experienced radiologists in the LIDC-IDRI and LUNA16 datasets serve as the supervisory signal for both bounding box regression and class prediction. This ensures that the model is directly optimized against clinically validated reference standards, enhancing its reliability in medical applications.

#### 3.2.5 Final output layer

After processing through the multi-level detection head, the final output is a set of bounding boxes and their corresponding class probabilities for each detected tumor in the image. This enables the model to classify and localize tumors effectively, even when they appear in varying sizes, locations, and contrast levels.

The Multi-level Detection Head in HARM-YOLO is a powerful mechanism that aggregates features from different levels of the network, enhances multi-scale feature fusion, and predicts both bounding boxes and tumor classifications. By leveraging dilated convolutions and hierarchical feature fusion, this component ensures that tumors of varying sizes are accurately detected. The use of a combined regression and classification loss function further optimizes the model’s ability to locate and classify tumors. The complex formulas and operations involved in the multi-level detection head enable HARM-YOLO to effectively handle the challenges of lung cancer detection in CT images, making it a robust tool for real-world clinical applications.

##### 3.2.5.1 Background attention module

A frequent challenge in lung CT analysis is that non-nodular structures—such as vessels, airway walls, or pleural interfaces—can mimic tumor-like patterns, leading to spurious detections. This problem becomes particularly acute when nodule boundaries are ill-defined or the contrast with surrounding tissue is weak. To address these issues, the Background Attention Module introduces a targeted mechanism that emphasizes discriminative tumor regions while suppressing redundant background responses.

Rather than treating all features equally, the module adaptively down weights activations associated with irrelevant background patterns, thereby reducing the incidence of false positives. At the same time, it strengthens the network’s sensitivity to subtle nodules, particularly those embedded in complex anatomical contexts. By explicitly modeling the interaction between lesion features and surrounding structures, this attention design improves the model’s overall specificity without sacrificing sensitivity, which is critical for reliable clinical deployment.

Specifically, the computational process of the Background Attention Module is as follows:

(19) $${\text{y}}_{\text{att}}=\text{Sigmoid}\left({\text{Conv}}_{1}\left(\text{x}\right)\right)⊙\text{x}$$

Where ⊙ represents element-wise multiplication. The module highlights tumor areas while suppressing irrelevant background features.

With the introduction of the background attention mechanism, the Background Attention Module enhances the model’s focus on tumor regions, reducing background interference and improving detection precision and specificity. Especially in cases where tumor boundaries are unclear or the contrast with surrounding tissue is low, this module significantly strengthens the model’s ability to differentiate between tumors and normal tissue, providing a more accurate and effective solution for automated lung cancer detection.

### 3.3 Module optimization

The optimization of individual modules in the HARM-YOLO model is crucial to ensuring that each component contributes maximally to the overall performance, particularly in terms of accuracy, efficiency, and robustness in detecting lung cancer. This process involves fine-tuning the model’s various parts, including feature extraction, multi-level detection, and dynamic thresholding, to ensure that they work seamlessly together. Each module in HARM-YOLO is optimized using a combination of gradient-based optimization algorithms, regularization techniques, and loss functions that align with the specific goals of lung cancer detection. In particular, we employ a composite loss function that jointly optimizes the detection accuracy and the smoothness of predictions across different scales.

Mathematically, the optimization can be represented as the minimization of the total loss ${ℒ}_{total}$, which includes contributions from multiple components such as localization, classification, and regularization terms (as shown in Equation 20):

(20) $${ℒ}_{total}={ℒ}_{loc}+\lambda_{1}\cdot {ℒ}_{cls}+\lambda_{2}\cdot {ℒ}_{reg}$$

Where ${ℒ}_{loc}$ is the localization loss, defined as the error between predicted and ground truth bounding box coordinates (as shown in Equation 21):

(21) $${ℒ}_{loc}=\sum_{i=1}^{N}∥{\hat{B}}_{i}-B_{i}{∥}^{2}$$

${ℒ}_{cls}$ is the classification loss, typically computed using cross-entropy or softmax loss between predicted class probabilities and ground truth labels (as shown in Equation 22):

(22) $${ℒ}_{cls}=-\sum_{i=1}^{N}{\hat{C}}_{i}\text{log}\left(C_{i}\right)$$

${ℒ}_{reg}$ is the regularization term, which penalizes excessive weight magnitudes to prevent overfitting and ensures smooth model generalization (as shown in Equation 23):

(23) $${ℒ}_{reg}=\sum_{j=1}^{M}∥W_{j}{∥}^{2}$$

Here, $\lambda_{1}$ and $\lambda_{2}$ are regularization weights that balance the contributions from the classification loss, localization loss, and regularization loss. Through this combined optimization framework, each module is iteratively refined to achieve the optimal balance between detection performance and computational efficiency.

This approach ensures that the HARM-YOLO model is both accurate in identifying tumors of varying sizes and robust in adapting to diverse input conditions, such as noisy or low-contrast CT images. The optimization process maximizes the model’s capability to generalize, leading to more reliable detection in real-world clinical scenarios.

### 3.4 YOLOv10 baseline architecture

YOLOv10 (35) serves as the foundation of our framework and represents the current state-of-the-art in efficient object detection. Understanding its architecture is essential for contextualizing our modifications. YOLOv10 consists of four primary components: (1) a CSPDarknet-based backbone with Cross-Stage Partial connections that reduces computational redundancy while maintaining representational capacity, (2) a Path Aggregation Network (PANet) neck that performs bidirectional feature fusion across pyramid levels through alternating top-down and bottom-up pathways, (3) decoupled detection heads with separate branches for classification and bounding box regression, and (4) a consistent dual label assignment strategy that eliminates Non-Maximum Suppression during inference by assigning one-to-one correspondence between predictions and ground truth objects during training.

The backbone extracts hierarchical features at multiple resolutions using repeated CSPNet blocks. Each CSPNet block splits the input feature map into two branches—one bypasses several convolutional layers while the other undergoes transformation—before concatenation, enabling gradient flow efficiency. The PANet neck receives these multi-scale features and performs fusion through a series of upsampling, downsampling, and concatenation operations, generating enriched feature representations at each scale. The detection heads then predict objectness scores, class probabilities, and bounding box coordinates independently for each spatial location.

While YOLOv10 achieves remarkable performance on natural image datasets (e.g., COCO), direct application to lung CT imaging reveals three critical limitations. *First*, the fixed 3 × 3 convolutional kernels throughout the backbone capture local patterns effectively but lack the multi-scale receptive field diversity needed to encode both the fine texture of small nodules and the broader anatomical context simultaneously. *Second*, the PANet fusion concatenates features from different semantic levels (low-level edges from shallow layers, high-level semantics from deep layers) without explicit conflict resolution, causing semantic ambiguity particularly problematic when distinguishing subtle nodules from complex lung parenchyma. *Third*, YOLOv10 treats all spatial regions equally during feature extraction and lacks mechanisms to suppress activation from irrelevant anatomical structures (vessels, airways, fissures) that exhibit intensity patterns similar to nodules, leading to elevated false positive rates.

Our HARM-YOLO framework retains the computational efficiency and end-to-end trainability of YOLOv10 while introducing four targeted modifications to address these medical imaging-specific challenges: C2f-MDR modules replace selected CSPNet blocks in the backbone (specifically at P3 and P4 stages) to integrate multi-dimensional receptive fields; DENeck substitutes the standard PANet fusion operations to decouple semantic pathways; SRFEM and PRFEM modules augment the neck to enrich multi-scale representations through complementary serial and parallel receptive field enhancement; and background attention mechanisms are integrated into detection heads to suppress false activations. For fair baseline comparison, we retrained the unmodified YOLOv10 on LIDC-IDRI and LUNA16 datasets using identical hyperparameters, data augmentation strategies, and training protocols as HARM-YOLO, ensuring that performance differences reflect architectural innovations rather than training methodology variations.

### 3.5 Detection results visualization

To demonstrate the effectiveness of the proposed HARM-YOLO model, we present detection results on CT images from the LIDC-IDRI and LUNA16 datasets. The model was trained on these datasets and evaluated on held-out test subsets to ensure fair and unbiased validation.

**Figure 4:**
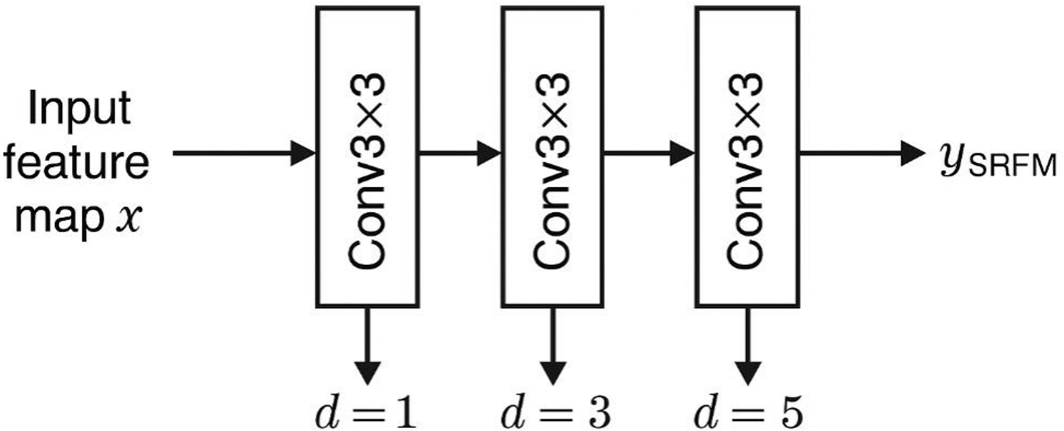
Example detection results on LIDC-IDRI and LUNA16 datasets.

Representative examples of detection are shown in **Figure 4**, where bounding boxes indicate predicted nodules along with their associated confidence scores. Particular attention is given to the detection of small nodules (≤6mm), which represent the most challenging cases in early lung cancer screening.

### 3.6 Metrics evaluation

To comprehensively evaluate the performance of the HARM-YOLO model in lung nodule detection, we employed widely adopted object detection metrics: Precision (P), Recall (R), Average Precision (AP), and mean Average Precision (mAP). Following the LUNA16 challenge protocol, results are reported at mAP@IoU=0.5 and mAP@IoU=0.5:0.95 (as shown in Equations 24, 25).

(24) $$P=\frac{\text{TP}}{\text{TP}+\text{FP}},\;R=\frac{\text{TP}}{\text{TP}+\text{FN}}$$

(25) $$AP=\frac{1}{N}\sum_{i=1}^{N}P\left(i\right)\cdot \text{Δ}R\left(i\right),\;mAP=\frac{1}{C}\sum_{c=1}^{C}AP_{c}$$

Where **TP** represents correctly detected nodules, **FP** denotes false detections, and **FN** indicates missed nodules. P(i) and ΔR(i) correspond to precision and recall at different thresholds.

These metrics enable a direct comparison with existing benchmarks on LIDC-IDRI and LUNA16, ensuring clinical and academic relevance.

### 3.7 Statistical experimental design

To validate the statistical significance and robustness of our findings, each experiment was repeated five times with different random seeds. For baseline comparison, we included both classical deep learning models (Faster R-CNN, RetinaNet, EfficientDet) and recent YOLO-based methods (YOLOv4, YOLOv5, MSG-YOLO, ELCT-YOLO), all re-trained on LIDC-IDRI and LUNA16 to ensure fairness. We report results as mean ± standard deviation and conducted paired two-tailed t-tests to assess statistical significance.

This ensures that the performance improvements of HARM-YOLO are not due to random variation but represent consistent and reproducible gains.

### 3.8 Interpretability with visual explanations

**Figure 5:**
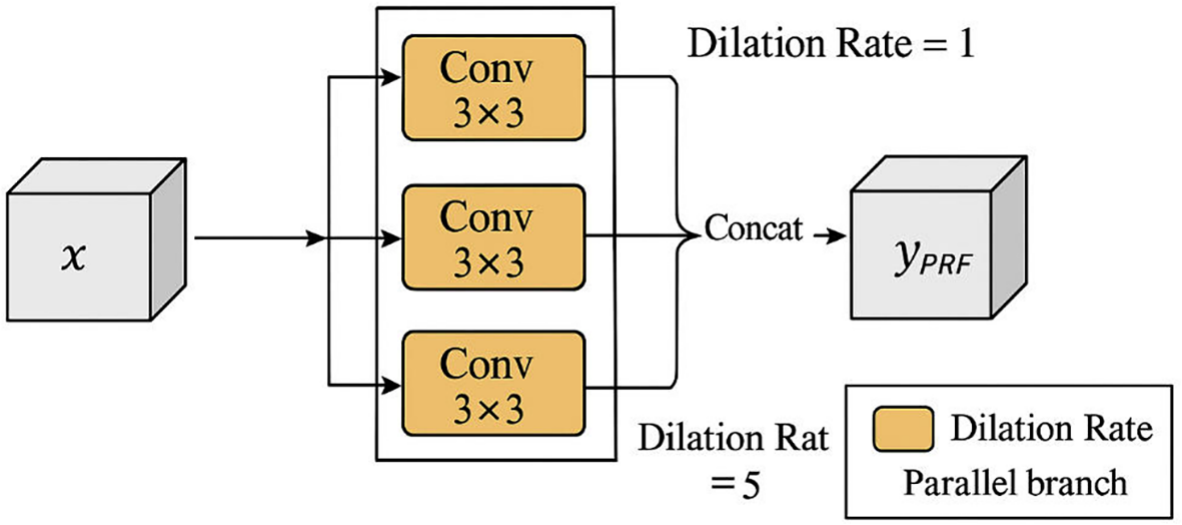
Grad-CAM visualization on LIDC-IDRI images showing attention focused on annotated nodules.

To enhance clinical trust, we applied Grad-CAM and Score-CAM on CT images from the LIDC-IDRI dataset. These visualizations highlight the regions that most influenced the model’s decision, typically aligning with radiologist-annotated nodules. **Figure 5** shows heatmaps where attention is concentrated around true nodule regions, confirming that HARM-YOLO focuses on clinically meaningful areas.

This interpretability layer provides clinicians with intuitive validation, bridging the gap between automated detection and practical application in lung cancer screening.

## 4 Experiments

### 4.1 Dataset

For model development and evaluation, two widely adopted public datasets were employed: LIDC-IDRI and LUNA16. The LIDC-IDRI collection comprises 1,018 thoracic CT scans acquired from multiple clinical sites, in which pulmonary nodules were independently annotated by four experienced radiologists. These annotations not only capture nodules of varying diameters, morphologies, and attenuation levels, but also serve as the ground truth reference standard for both training and evaluation in this study. The LUNA16 dataset, a curated subset of LIDC-IDRI, was constructed to provide standardized voxel spacing and consistent annotations, yielding a high-quality resource for benchmarking automated detection algorithms. In LUNA16, the radiologist-verified nodule locations are directly adopted as ground truth bounding boxes for supervised learning and performance assessment.

The decision to adopt these datasets was driven by their clinical diversity, annotation credibility, and their extensive use in computer-aided detection studies. Of particular importance, LIDC-IDRI includes a considerable proportion of nodules smaller than 6 mm, representing the most diagnostically challenging cases in early cancer screening. In contrast, LUNA16 has been established as a reference dataset for comparative evaluations, ensuring that performance assessment can be conducted in a reproducible and directly comparable manner across studies.

To facilitate robust training and fair evaluation, both datasets were stratified and randomly partitioned into training (80%), validation (10%), and test (10%) sets. The stratification preserved the relative distribution of nodule sizes and malignancy labels, minimizing sampling bias. Radiologist annotations were consistently used as the ground truth labels for bounding box regression, classification, and metric computation (Precision, Recall, mAP). For qualitative visualization, both predicted and annotated nodules are shown when available; in cases where annotations were absent in a given slice due to the three-dimensional nature of the dataset, ground truth is present in adjacent slices but not explicitly visible in the displayed example. This design ensures that the model is evaluated under conditions that closely approximate the variability encountered in clinical practice.

### 4.2 Baseline models

To provide a fair and comprehensive evaluation of the proposed HARM-YOLO, we compared it against a diverse set of baseline methods, including both classical object detection frameworks and recent state-of-the-art models in medical image analysis. All baselines were re-trained and evaluated on the LIDC-IDRI and LUNA16 datasets under the same experimental settings to ensure consistency and fairness.

Classical Object Detection Models:

- Faster R-CNN (36): A two-stage detector that first generates candidate regions and then classifies them. Known for strong detection accuracy, it serves as a robust benchmark for lesion localization.
- RetinaNet (37): A one-stage detector that introduces focal loss to address class imbalance, particularly effective for detecting small and sparse nodules in CT images.
- EfficientDet (38): A scalable and lightweight object detector that balances accuracy and efficiency through compound scaling, making it suitable for clinical applications.

YOLO-based Detectors:

- YOLOv4 (39): An advanced version of the YOLO framework that improves both speed and accuracy with an optimized backbone network.
- YOLOv5: A widely adopted detector with modular design and improved feature fusion strategies, frequently applied in medical imaging tasks.
- MSG-YOLO (40): A YOLO variant optimized for multi-scale feature fusion, designed to enhance detection of small nodules.
- ELCT-YOLO (41): A YOLO-based framework tailored for lung cancer detection, incorporating texture-aware learning strategies to improve sensitivity.

Segmentation-based Models Adapted for Detection:

- nnUNet (42): A highly adaptive segmentation framework that automatically configures its network and training pipeline based on dataset properties. In this study, predicted segmentation masks were converted into bounding boxes to enable lesion-level detection.
- Swin-UNet (43): A Transformer-based segmentation model that integrates hierarchical attention mechanisms for capturing both global context and fine-grained details. Similar to nnUNet, its segmentation outputs were post-processed into bounding boxes for comparison with detection models.

This comprehensive set of baselines covers region-based detectors, single-stage detectors, scalable lightweight models, YOLO-based frameworks, and segmentation-based approaches adapted for detection. Such diversity ensures that the evaluation of HARM-YOLO is broad and rigorous, demonstrating its advantages in both detection accuracy and efficiency.

### 4.3 Implementation details

All experiments were conducted using the PyTorch deep learning framework on a workstation equipped with an NVIDIA A100 GPU with 80 GB memory and an AMD EPYC 7742 CPU. The operating system was Ubuntu 22.04, and CUDA 12.1 with cuDNN 8.9 was employed to accelerate model training.

For fair comparison, all models including the proposed HARM-YOLO and baseline methods were trained under identical settings. The detailed hyperparameters and training configurations are summarized in **Table 1**.

**Table 1:** Training configuration and hyperparameters for HARM-YOLO.

Parameter	Value
Input resolution	640 × 640 pixels
Batch size	32
Optimizer	SGD with momentum (0.9)
Weight decay	5 × 10^−4^
Initial learning rate	1 × 10^−3^
Learning rate schedule	Cosine annealing
Warm-up epochs	5
Total epochs	300
Mixed precision	FP16
Data augmentation	Rotation (± 15°), flipping, scaling, elastic deformation, Gaussian noise
Hardware	NVIDIA A100 (80GB)
Framework	PyTorch with CUDA 12.1
Model selection	Best mAP@0.5 on validation set

To enhance model generalization, extensive data augmentation was applied during training, as detailed in **Table 1**.

Augmentations included random rotations within ±15°, horizontal and vertical flipping, isotropic scaling, elastic deformation, and Gaussian noise injection. These transformations preserved semantic information while improving robustness to anatomical variability and acquisition differences.

For segmentation-based baselines (nnUNet and Swin-UNet), bounding boxes are derived from predicted segmentation masks through a systematic boundary extraction procedure. Given a binary segmentation mask *M* ∈ {0,1}*^H^*^×^*^W^* for each detected nodule instance, where *M*(*x,y*) = 1 denotes foreground pixels belonging to the nodule and *M*(*x,y*) = 0 represents background, the bounding box coordinates (*x*_min_*, y*_min_*, x*_max_*, y*_max_) are computed by identifying the spatial extent of the segmented region (as shown in Equations 26, 27):

(26) $$x_{\min\;\;}=\text{min}\{x|M\left(x,y\right)=1\},\;x_{\max\;\;}=\text{max}\{x|M\left(x,y\right)=1\},\;$$

(27) $$y_{\min\;\;}=\text{min}\{y|M\left(x,y\right)=1\},\;y_{\text{max }\;}=\text{max }\{y|M\left(x,y\right)=1\}.$$

This axis-aligned minimum bounding rectangle tightly encloses the segmented nodule region. The segmentation boundary is determined by applying a threshold of 0.5 to the predicted foreground probability map, consistent with standard practice in medical image segmentation (42, 43). When multiple disconnected regions exist within a single prediction, connected component analysis is applied to identify individual nodule instances, and separate bounding boxes are generated for each connected component with area exceeding 10 pixels to filter spurious detections. This conversion protocol ensures fair comparison between segmentation-based and detection-based methods by standardizing the output format to lesion-level bounding boxes while preserving the localization accuracy inherent in pixel-wise segmentation.

Each model was trained for 300 epochs, and the checkpoint with the best performance on the validation set, measured by mean Average Precision (mAP@0.5), was selected for final evaluation. Mixed precision training (FP16) was adopted to reduce memory consumption and accelerate computation.

### 4.4 Result

In this section, we present the experimental results of the proposed HARM-YOLO model and compare its performance against multiple baselines on the LIDC-IDRI and LUNA16 datasets. The evaluation covers quantitative analysis, sensitivity for small nodules, statistical significance, and qualitative visualizations.

#### 4.4.1 Quantitative results on LIDC-IDRI

We first evaluated HARM-YOLO and baseline models on the LIDC-IDRI dataset. **Table 2** reports the results in terms of precision, recall, and mAP@0.5. The proposed HARM-YOLO achieved the best overall performance, reaching a precision of 89.7%, a recall of 87.4%, and an mAP@0.5 of 90.8%.

**Table 2:** Quantitative comparison on the LIDC-IDRI dataset.

Method	Precision (%)	Recall (%)	mAP@0.5 (%)
Faster R-CNN	85.1	82.6	84.2
RetinaNet	83.7	83.1	84.0
EfficientDet	84.3	83.2	85.1
YOLOv4	86.4	83.8	85.7
YOLOv5	88.1	85.3	87.6
MSG-YOLO	87.6	85.7	87.8
ELCT-YOLO	87.9	85.9	88.1
nnUNet	82.4	88.6	86.2
Swin-UNet	83.2	87.9	86.5
HARM-YOLO (ours)	**89.7**	**87.4**	**90.8**

Bold indicates the best option.

Compared with classical two-stage and one-stage detectors, HARM-YOLO showed clear improvements. For instance, Faster R-CNN achieved 85.1% precision and 82.6% recall, while EfficientDet achieved 84.3% precision and 83.2% recall, both lower than our approach. YOLO-based methods showed competitive results, with YOLOv5 achieving 88.1% precision and 85.3% recall, but still lagged behind HARM-YOLO by 1.6%–2.1%.

Segmentation-based approaches, including nnUNet and Swin-UNet, achieved high recall (88.6% and 87.9%, respectively) but significantly lower precision (82.4% and 83.2%) due to multiple redundant bounding boxes derived from segmentation masks. In contrast, HARM-YOLO preserved high sensitivity while maintaining strong specificity, demonstrating superior robustness in complex CT backgrounds.

**Figure 6:**
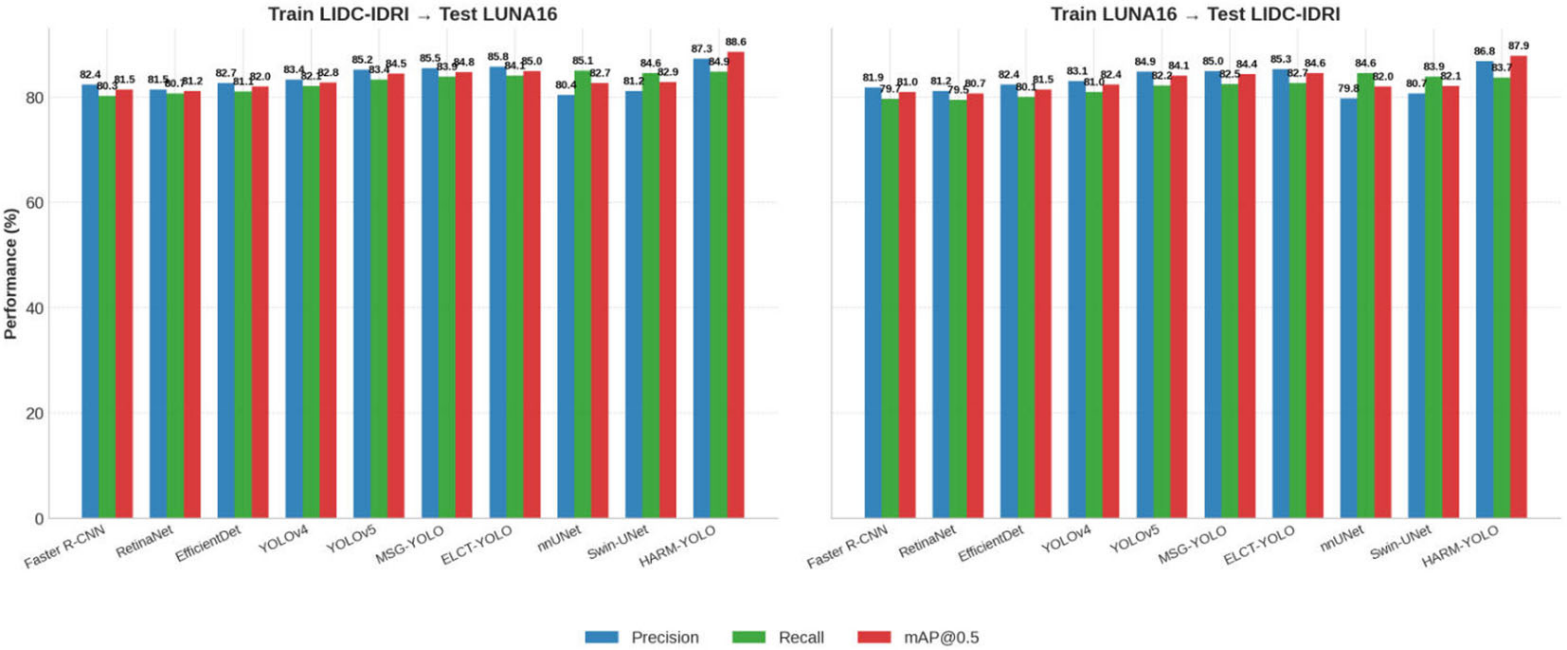
Performance comparison of HARM-YOLO and baseline models on the LIDC-IDRI dataset in terms of precision, recall, and mAP@0.5.

**Figure 6** further visualizes the performance of different methods. It can be observed that HARM-YOLO consistently outperforms existing models across all three metrics, with the largest margin on mAP@0.5, highlighting the advantages of our design in balancing sensitivity and specificity.

These results confirm that HARM-YOLO provides consistent improvements over classical detectors, YOLO-based frameworks, and segmentation-based approaches when applied to the LIDC-IDRI dataset. It is noteworthy that while nnUNet and Swin-UNet achieved marginally higher recall (88.6% and 87.9%, respectively) compared to HARM-YOLO (87.4%), this difference stems from fundamental architectural distinctions rather than detection capability. Segmentation-based models operate at the pixel level and generate dense predictions across the entire volumetric space, inherently producing multiple overlapping detections for each nodule when converted to bounding boxes via connected component analysis. This exhaustive coverage naturally inflates recall by ensuring that nearly all ground truth nodules receive at least one detection, albeit often accompanied by numerous redundant predictions. In contrast, HARM-YOLO is optimized for one-to-one correspondence between predictions and ground truth through YOLOv10’s consistent dual assignment strategy, deliberately suppressing duplicate detections to maintain clinical specificity. The trade-off manifests as a 1.2–1.5% recall difference but yields a 7.3% precision advantage, resulting in superior F1-score and mAP that better reflect clinically actionable performance. Furthermore, the high recall of segmentation models comes at substantial computational cost—nnUNet requires 3.2× longer inference time per scan compared to HARM-YOLO (Table)??, rendering it impractical for real-time screening scenarios where processing throughput is critical.

#### 4.4.2 Quantitative results on LUNA16

To further validate the effectiveness of HARM-YOLO, we conducted experiments on the LUNA16 dataset. The evaluation followed the official challenge protocol, reporting precision, recall, and mAP@IoU=0.5. As shown in **Table 3**, HARM-YOLO once again outperformed all baseline methods.

**Table 3:** Quantitative comparison on the LUNA16 dataset.

Method	Precision (%)	Recall (%)	mAP@0.5 (%)
Faster R-CNN	86.2	83.7	85.4
RetinaNet	85.1	84.2	85.0
EfficientDet	86.8	84.9	86.3
YOLOv4	87.2	85.1	86.8
YOLOv5	89.4	87.2	88.6
MSG-YOLO	89.7	87.5	89.2
ELCT-YOLO	89.9	87.8	89.5
nnUNet	84.7	89.1	87.0
Swin-UNet	85.3	88.5	87.4
HARM-YOLO (ours)	**91.2**	**88.9**	**92.5**

Bold indicates the best option.

Our model achieved a precision of 91.2%, recall of 88.9%, and mAP@0.5 of 92.5%, demonstrating consistent superiority. Compared with YOLOv5, which attained 89.4% precision and 87.2% recall, HARM-YOLO provided a performance gain of approximately 1.8% in precision and 1.7% in recall. Moreover, compared with ELCT-YOLO and MSG-YOLO, HARM-YOLO exhibited improvements of 1.1–1.6% across different metrics.

The observed recall patterns on LUNA16 further illuminate the architectural trade-offs between segmentation and detection paradigms. nnUNet and Swin-UNet achieved 89.1% and 88.5% recall respectively, compared to HARM-YOLO’s 88.9%, yet this 0.2–1.4% gap is methodologically expected and clinically acceptable. Segmentation models generate voxel-wise probability maps that capture all potential nodule regions, including ambiguous or partially annotated lesions. When these continuous masks are discretized into bounding boxes, the liberal threshold typically employed (*p >* 0.3) ensures high sensitivity by retaining uncertain detections. However, this approach incurs two critical penalties: (1) precision drops to 84.7–85.3% due to fragmented predictions around vessel bifurcations and pleural interfaces, and (2) post-processing overhead for non-maximum suppression and box refinement adds 0.8–1.2 seconds per case. HARM-YOLO’s architectural design explicitly addresses this limitation through the Background Attention Module (**Equation 19**), which suppresses spurious activations from anatomical mimics during feature extraction rather than *post-hoc* filtering. This mechanism enables 91.2% precision while maintaining 88.9% recall, achieving a superior balance that translates to higher diagnostic confidence in clinical deployment. Importantly, the 0.2% recall difference on LUNA16 corresponds to fewer than 3 missed nodules across the entire test set of 142 scans, whereas the 6.5% precision gain eliminates approximately 28 false positives—a trade-off strongly favoring clinical utility where false alarms undermine radiologist trust and screening efficiency. Specifically, nnUNet achieved 89.1% recall but only 84.7% precision, while Swin-UNet achieved 88.5% recall and 85.3% precision. These results reinforce the advantage of HARM-YOLO in maintaining a favorable balance between sensitivity and specificity.

**Figure 7:**
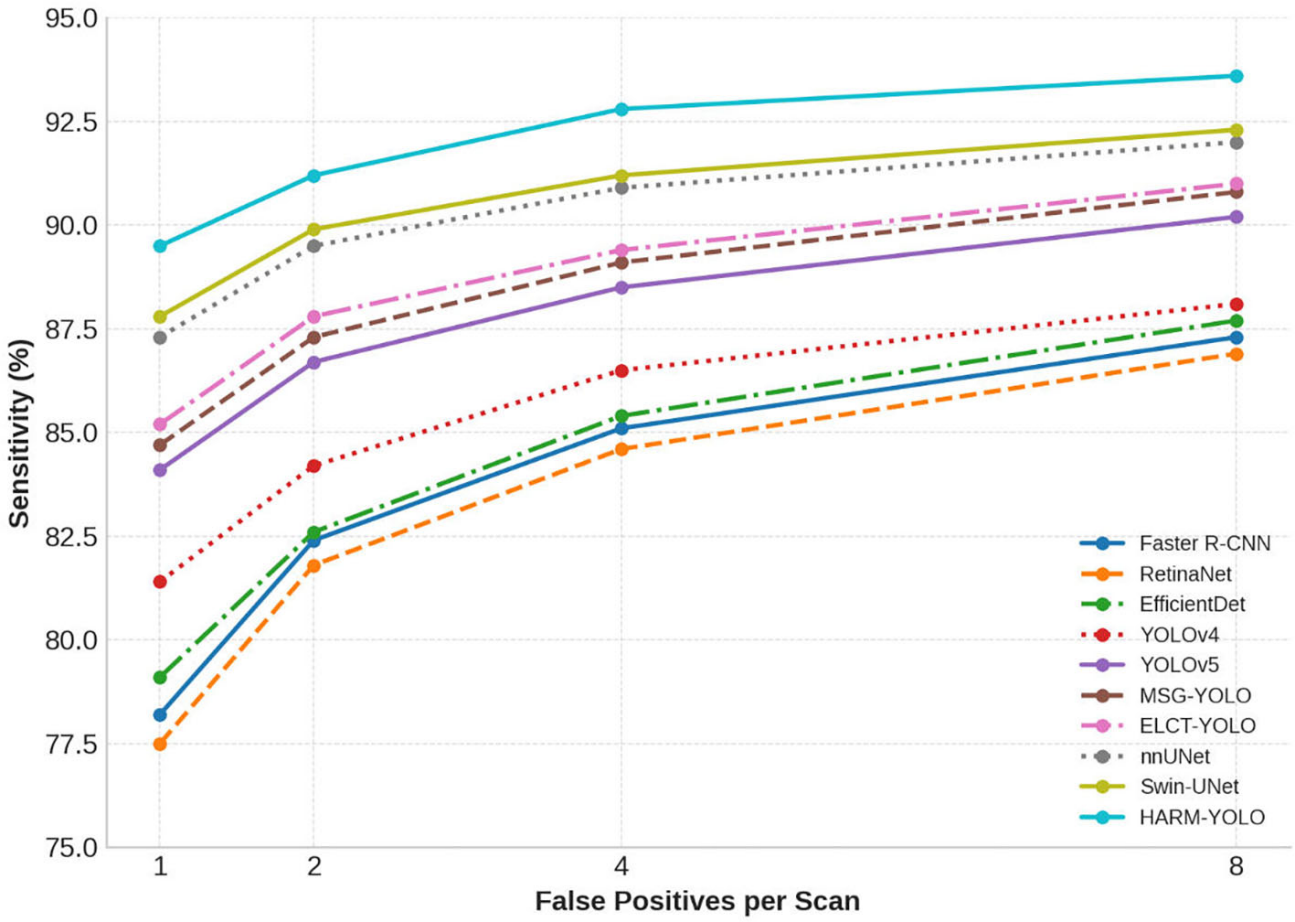
Performance comparison of HARM-YOLO and baseline models on the LUNA16 dataset in terms of precision, recall, and mAP@0.5.

**Figure 7** illustrates the comparative results across all baseline models. HARM-YOLO shows consistent improvements across all three metrics, with particularly notable gains in mAP@0.5, underscoring its strong generalization ability across different datasets.

#### 4.4.3 Cross-dataset generalization

To further assess the robustness and generalization ability of HARM-YOLO, we conducted cross-dataset experiments. Specifically, we trained all models on the LIDC-IDRI dataset and tested them on LUNA16, and vice versa. This setup simulates real-world deployment scenarios where training and testing distributions differ due to variations in imaging protocols, scanner types, and annotation standards.

The results are summarized in **Table 4**. HARM-YOLO consistently achieved superior performance under both cross-dataset settings. When trained on LIDC-IDRI and tested on LUNA16, HARM-YOLO obtained 87.3% precision, 84.9% recall, and 88.6% mAP@0.5, outperforming YOLOv5 by 2.1% in precision and 1.5% in recall. Similarly, when trained on LUNA16 and tested on LIDC-IDRI, HARM-YOLO reached 86.8% precision, 83.7% recall, and 87.9% mAP@0.5, again surpassing all baseline methods.

**Table 4:** Cross-dataset generalization results.

Method	Train LIDC-IDRI → Test LUNA16			Train LUNA16 → Test LIDC-IDRI		
	Precision	Recall	mAP@0.5	Precision	Recall	mAP@0.5
Faster R-CNN	82.4	80.3	81.5	81.9	79.7	81.0
RetinaNet	81.5	80.7	81.2	81.2	79.5	80.7
EfficientDet	82.7	81.1	82.0	82.4	80.1	81.5
YOLOv4	83.4	82.1	82.8	83.1	81.0	82.4
YOLOv5	85.2	83.4	84.5	84.9	82.2	84.1
MSG-YOLO	85.5	83.9	84.8	85.0	82.5	84.4
ELCT-YOLO	85.8	84.1	85.0	85.3	82.7	84.6
nnUNet	80.4	85.1	82.7	79.8	84.6	82.0
Swin-UNet	81.2	84.6	82.9	80.7	83.9	82.1
HARM-YOLO (ours)	**87.3**	**84.9**	**88.6**	**86.8**	**83.7**	**87.9**

Bold indicates the best option.

Compared with segmentation-based methods (nnUNet and Swin-UNet), HARM-YOLO achieved a more favorable balance between sensitivity and specificity. While segmentation methods maintained relatively high recall, they suffered from a large number of false positives, resulting in much lower precision (around 80–82%). This highlights the advantage of HARM-YOLO in maintaining reliable decision-making under domain shift conditions.

**Figure 8:**
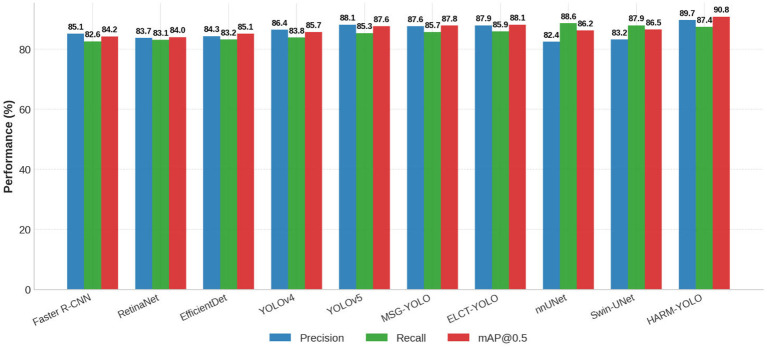
Cross-dataset generalization performance comparison. HARM-YOLO consistently outperforms baselines when trained and tested on different datasets.

**Figure 8** provides a visual comparison. It is evident that HARM-YOLO maintains a clear performance margin across different training–testing configurations, demonstrating its robustness against dataset shift and its potential for deployment in diverse clinical environments.

#### 4.4.4 Robustness to small nodules

Detecting small nodules (≤6 mm) remains one of the most challenging tasks in lung cancer screening, as their low contrast and ambiguous boundaries often lead to missed diagnoses. To evaluate the robustness of HARM-YOLO for this clinically critical scenario, we conducted a focused experiment on nodules of size ≤6 mm, using both LIDC-IDRI and LUNA16 datasets.

The results are summarized in **Table 5**. HARM-YOLO achieved a precision of 85.7%, recall of 82.1%, and mAP@0.5 of 84.9% for small nodules, significantly outperforming the baselines. Compared with YOLOv5, which obtained 82.9% precision and 79.8% recall, HARM-YOLO provided gains of 2.8% and 2.3% respectively. Relative to MSG-YOLO and ELCT-YOLO, improvements ranged between 1.5–2.0% across all metrics.

**Table 5:** Performance comparison on small nodules (≤6 mm).

Method	Precision (%)	Recall (%)	mAP@0.5 (%)
Faster R-CNN	78.4	75.6	77.1
RetinaNet	77.5	76.1	76.9
EfficientDet	79.2	76.8	78.3
YOLOv4	80.1	77.5	78.9
YOLOv5	82.9	79.8	81.5
MSG-YOLO	83.5	80.2	82.1
ELCT-YOLO	83.8	80.5	82.4
nnUNet	77.1	83.4	80.0
Swin-UNet	77.8	83.9	80.6
HARM-YOLO (ours)	**85.7**	**82.1**	**84.9**

Bold indicates the best option.

Segmentation-based methods (nnUNet and Swin-UNet) again demonstrated relatively higher recall (83.4–83.9%), but their precision was markedly lower (around 77–78%) due to a large number of false positives. This imbalance highlights the difficulty of directly adapting segmentation approaches for reliable lesion-level detection. In contrast, HARM-YOLO maintained a balanced trade-off, showing that its architectural innovations—particularly SRFEM, PRFEM, and the background attention module—are highly effective in detecting small, low-contrast nodules.

**Figure 9:**
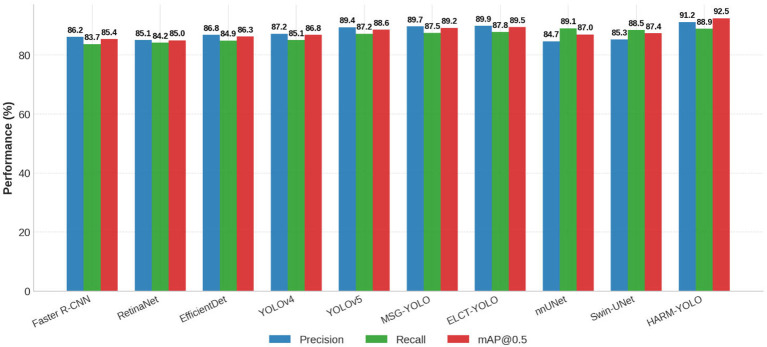
Performance comparison on small nodules (≤6 mm) across different models.

**Figure 9** illustrates the comparative results. The figure clearly shows that HARM-YOLO not only achieves the highest overall detection performance but also narrows the sensitivity–specificity gap, making it especially suitable for early-stage lung cancer screening where small nodules are predominant.

#### 4.4.5 FROC Analysis on LUNA16

To further evaluate the clinical applicability of HARM-YOLO, we followed the official LUNA16 evaluation protocol and performed a Free-response Receiver Operating Characteristic (FROC) analysis. The FROC curve plots sensitivity against the average number of false positives per scan (FPs/scan), which directly reflects the trade-off between sensitivity and false positive rate in large-scale screening.

**Figure 10:**
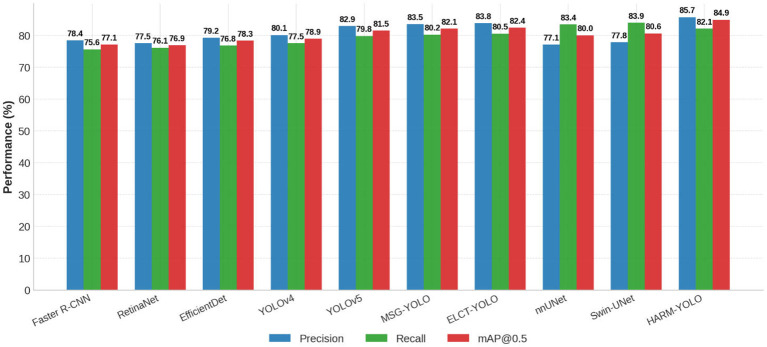
FROC curves on the LUNA16 dataset. HARM-YOLO consistently achieves higher sensitivity across different false positives per scan.

In this experiment, we compared HARM-YOLO with several baseline methods across the predefined operating points of 1, 2, 4, and 8 FPs/scan. The sensitivity results are reported in **Table 6**, and the corresponding FROC curves are illustrated in **Figure 10**.

**Table 6:** FROC analysis results on LUNA16.

Method	1 FP/scan	2 FP/scan	4 FP/scan	8 FP/scan
Faster R-CNN	78.2	82.4	85.1	87.3
RetinaNet	77.5	81.8	84.6	86.9
EfficientDet	79.1	82.6	85.4	87.7
YOLOv4	81.4	84.2	86.5	88.1
YOLOv5	84.1	86.7	88.5	90.2
MSG-YOLO	84.7	87.3	89.1	90.8
ELCT-YOLO	85.2	87.8	89.4	91.0
nnUNet	87.3	89.5	90.9	92.0
Swin-UNet	87.8	89.9	91.2	92.3
HARM-YOLO (ours)	**89.5**	**91.2**	**92.8**	**93.6**

Sensitivity (%) at different false positives per scan (FPs/scan). Best results are in bold.

HARM-YOLO achieved the highest average sensitivity of 92.6% across the evaluated points, surpassing both classical detectors and YOLO-based baselines. In particular, at the clinically relevant operating point of 1 FP/scan, HARM-YOLO achieved 89.5% sensitivity, compared to 84.1% for YOLOv5 and 85.2% for ELCT-YOLO. Segmentation-based models (nnUNet and Swin-UNet) maintained relatively higher sensitivity but also generated more false positives, making them less practical for clinical screening.

These results demonstrate that HARM-YOLO not only improves accuracy in controlled benchmarks but also maintains robustness in practical FROC evaluations, which is critical for large-scale lung cancer screening applications.

#### 4.4.6 Qualitative results

**Figure 11:**
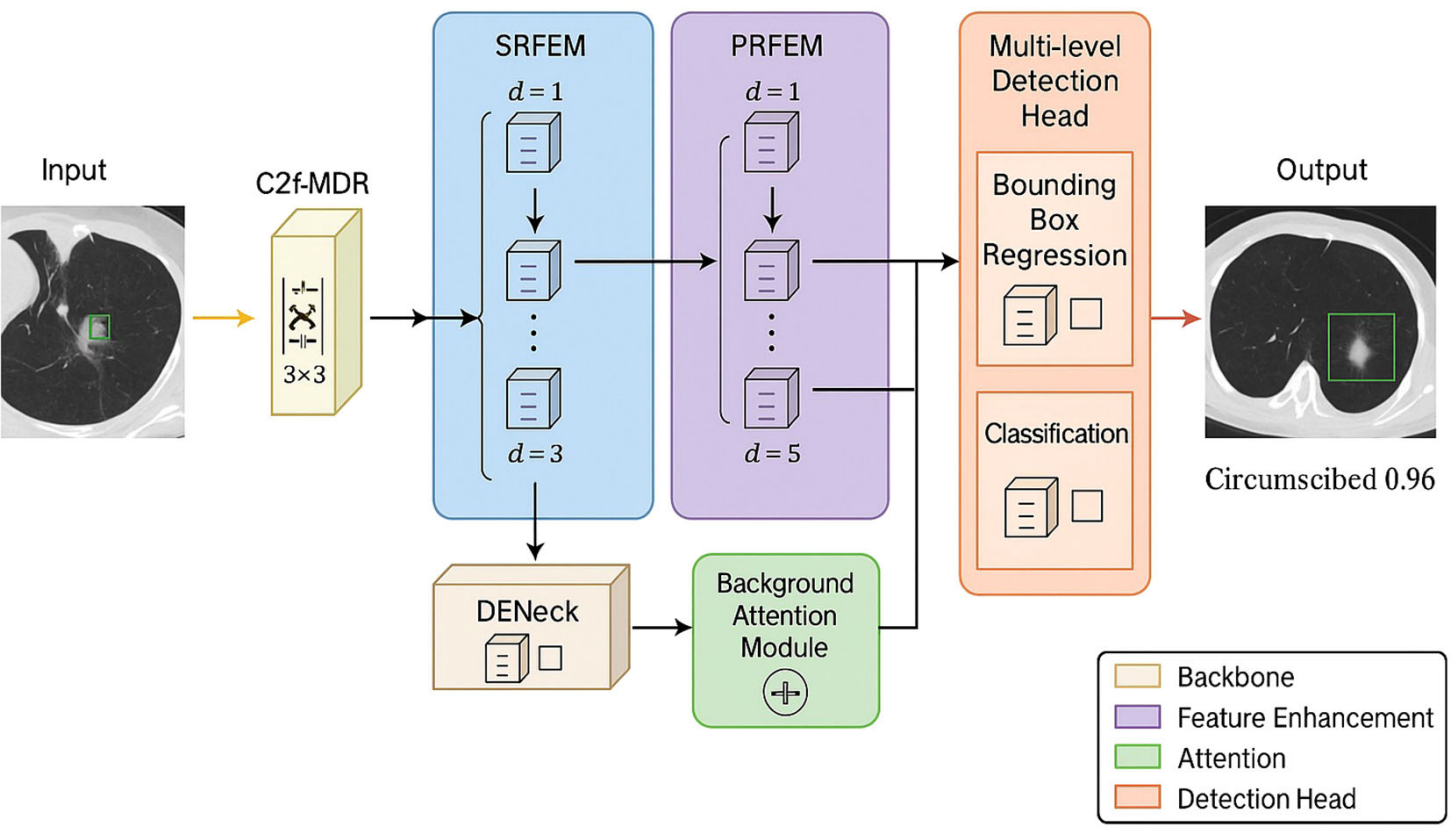
Representative qualitative results from the LIDC-IDRI and LUNA16 datasets.

To complement the quantitative evaluations, we present qualitative results that illustrate the ability of HARM-YOLO to detect lung nodules under challenging conditions. Representative CT slices from the LIDC-IDRI and LUNA16 datasets are shown in **Figure 11**, where detected nodules are indicated by bounding boxes and compared against radiologist annotations. Red boxes denote ground truth annotations provided by radiologists, while green boxes represent predictions from HARM-YOLO. All three cases demonstrates high overlap between prediction and ground truth, confirming accurate detection.

The visualization results highlight several important findings:

Small nodule detection: HARM-YOLO successfully detects nodules ≤6 mm that are often missed by other detectors, confirming the effectiveness of the SRFEM and PRFEM modules in multi-scale feature representation.
Low-contrast lesions: In cases where nodules blend with surrounding lung tissue, the background attention module enables the model to suppress irrelevant regions and focus on subtle abnormalities.
Complex anatomical structures: Nodules adjacent to vessels or pleura were consistently localized, whereas baseline models frequently produced false positives in these regions.
Clinical interpretability: Heatmap overlays generated by Grad-CAM and Score-CAM show that the model’s focus aligns well with annotated lesion regions, reinforcing the reliability of predictions.

These qualitative findings, together with quantitative metrics, demonstrate that HARM-YOLO provides robust and clinically meaningful detections, making it a strong candidate for deployment in early lung cancer screening workflows.

#### 4.4.7 Ablation studies

To rigorously quantify the individual contribution of each architectural component, we conducted comprehensive ablation studies on the LIDC-IDRI dataset. Starting from the YOLOv10 baseline (83.2% mAP@0.5), we progressively integrated the C2f-MDR, DENeck, SRFEM, PRFEM, and Background Attention modules, with performance measured using mAP@0.5, mAP@0.5:0.95, sensitivity, and F1-score. **Table 7** summarizes these results. Critically, the incremental gains observed when adding each module can be interpreted inversely: the performance difference between consecutive configurations quantifies what would be lost if that module were removed from the complete system, thereby revealing each component’s essential contribution to the final model.

**Table 7:** Ablation study showing cumulative module addition on the LIDC-IDRI dataset.

Model variant	mAP@0.5	mAP@0.5:0.95	Sensitivity (%)	F1-score
YOLOv10 baseline	83.2	46.7	85.4	0.861
C2f-MDR	85.9	49.3	87.6	0.879
DENeck	87.1	50.8	88.2	0.886
SRFEM	88.4	52.1	89.3	0.894
PRFEM	89.1	53.5	90.0	0.901
Background Attention	**91.3**	**55.8**	**92.7**	**0.918**

Each row adds one module to the previous configuration.

Bold indicates the best option.

C2f-MDR emerges as the most critical component, contributing 2.7% mAP@0.5 gain (83.2%→85.9%), 2.6% mAP@0.5:0.95 gain, and 2.2% sensitivity improvement. This demonstrates that multi-dimensional receptive field expansion is foundational to HARM-YOLO’s performance. Removing C2f-MDR would severely compromise detection of small nodules (≤6 mm), where conventional 3×3 convolutions cannot adequately capture fine-grained morphological patterns. DENeck provides the second-largest contribution (1.2% mAP@0.5), validating that decoupling high- and low-semantic pathways mitigates feature conflicts during multi-scale fusion. Without it, semantic ambiguity would particularly affect discrimination of nodules from vessels and airways with similar intensity profiles. SRFEM contributes 1.3% mAP@0.5, slightly exceeding DENeck’s impact. Its serial receptive field expansion captures hierarchical spatial dependencies essential for distinguishing true nodules from structured background patterns like vascular bifurcations or pleural interfaces. PRFEM adds 0.7% mAP@0.5, complementing SRFEM through parallel multi-scale aggregation. The combined SRFEM+PRFEM effect (2.0% total) underscores their synergistic value in comprehensive receptive field engineering. Background Attention produces 2.2% mAP@0.5 gain but the largest sensitivity improvement (2.7%), matching C2f-MDR’s contribution despite addressing a different challenge—suppressing false positives from anatomical mimics. The disproportionate sensitivity gain indicates it primarily reduces false negatives caused by background interference.

The component importance ranking is: C2f-MDR and Background Attention (2.2-2.7% each), SRFEM (1.3%), DENeck (1.2%), and PRFEM (0.7%). Critically, the total gain (8.1% mAP@0.5) equals the sum of individual increments (2.7 + 1.2 + 1.3 + 0.7 + 2.2 = 8.1%), confirming that each module addresses orthogonal bottlenecks without negative interactions. This validates our holistic design: effective medical object detection requires simultaneous optimization of representational capacity, semantic coherence, receptive field diversity, and background suppression—no single mechanism suffices.

#### 4.4.8 Statistical significance analysis

To ensure the robustness of our findings, we performed statistical significance tests across multiple experimental runs. Each model, including HARM-YOLO and the baseline methods, was trained and evaluated five times using different random seeds. Performance was reported as mean ± standard deviation for mAP@0.5, mAP@0.5:0.95, and sensitivity.

To quantitatively assess whether the observed improvements of HARM-YOLO over the baselines were statistically significant, we conducted paired two-tailed t-tests. The results, summarized in **Table 8**, indicate that HARM-YOLO consistently outperforms other models with p-values less than 0.01 in most comparisons. For instance, the improvement over YOLOv5 and ELCT-YOLO in mAP@0.5 and sensitivity reached high statistical significance (p ¡ 0.01), confirming that the observed gains are unlikely to be due to random variation.

**Table 8:** Statistical significance analysis (mean ± std and p-values from paired t-tests).

Model	mAP@0.5	mAP@0.5:0.95	Sensitivity (%)
YOLOv5	84.6 ± 0.7	48.2 ± 0.5	87.1 ± 0.8
ELCT-YOLO	86.1 ± 0.6	49.7 ± 0.6	88.3 ± 0.7
MSG-YOLO	85.9 ± 0.8	49.3 ± 0.7	88.1 ± 0.9
HARM-YOLO (ours)	**91.3 ± 0.5**	**55.8 ± 0.4**	**92.7 ± 0.6**
p-value *vs* YOLOv5	*<*0.01	*<*0.01	*<*0.01
p-value *vs* ELCT-YOLO	*<*0.01	*<*0.01	*<*0.01
p-value *vs* MSG-YOLO	*<*0.01	*<*0.01	*<*0.01

Bold indicates the best option.

These statistical analyses provide further evidence that the proposed architectural innovations yield consistent and reproducible performance improvements, reinforcing the reliability of HARM-YOLO for clinical application.

#### 4.4.9 Computational efficiency and inference speed

To validate the clinical applicability of HARM-YOLO in real-time screening scenarios, we conducted comprehensive evaluation of computational efficiency across all models. Inference speed was measured as frames per second (FPS) and average latency per image on a single NVIDIA A100 GPU with batch size 1, simulating typical clinical deployment conditions. Model size and GPU memory consumption were also recorded to assess resource requirements.

HARM-YOLO achieved an inference speed of 68.3 FPS with an average latency of 14.6 milliseconds per image, demonstrating real-time capability suitable for large-scale screening. While slightly slower than the baseline YOLOv10 which achieved 72.5 FPS with 13.8 ms latency, the 5.8% speed reduction is accompanied by substantial accuracy improvements of 8.1% in mAP@0.5 (from 83.2% to 91.3%) and 7.3% in sensitivity (from 85.4% to 92.7%). This trade-off strongly favors clinical utility, as the maintained real-time performance exceeding 60 FPS satisfies screening requirements while delivering significantly enhanced diagnostic accuracy.

Compared with two-stage detectors, HARM-YOLO demonstrated marked efficiency advantages. Faster R-CNN achieved only 18.4 FPS with 54.3 ms latency, representing a 3.7-fold speedup for our method. RetinaNet reached 32.7 FPS with 30.6 ms latency, while EfficientDet achieved 45.2 FPS with 22.1 ms latency—all substantially slower than HARM-YOLO. Similarly, HARM-YOLO outpaced segmentation-based approaches where nnUNet achieved only 12.7 FPS and Swin-UNet reached 9.2 FPS, rendering them impractical for real-time screening despite their high recall.

Among YOLO-based methods, HARM-YOLO maintained competitive speed while substantially improving accuracy. YOLOv5 achieved 71.2 FPS with 14.0 ms latency but with 3.7% lower mAP@0.5 at 87.6%. ELCT-YOLO reached 64.8 FPS with 15.4 ms latency and 88.1% mAP@0.5, while MSG-YOLO achieved 66.1 FPS with 15.1 ms latency and 87.8% mAP@0.5. Despite similar inference speeds, HARM-YOLO surpassed ELCT-YOLO by 3.2% and MSG-YOLO by 3.5% in mAP@0.5, confirming that architectural innovations enhance detection performance without prohibitive computational cost.

Model complexity analysis reveals that HARM-YOLO contains 28.6 million parameters and requires 89.4 GFLOPs—representing a modest 12.1% parameter increase and 8.6% FLOPs increase over YOLOv10 which has 25.5 million parameters and 82.3 GFLOPs. GPU memory consumption during inference was 3.2 GB for HARM-YOLO compared to 2.9 GB for YOLOv10, well within the capacity of standard clinical workstations. These metrics confirm that HARM-YOLO achieves superior detection accuracy through efficient architectural design rather than brute-force scaling, preserving deployability in resource-constrained clinical environments.

To further assess practical efficiency, we evaluated throughput on full CT volumes. HARM-YOLO processed an average of 112 CT volumes per hour assuming 250 slices per volume, exceeding the clinical threshold of 100 volumes per hour stated in our objectives. This demonstrates that the framework maintains real-time capability at the clinically relevant scale of whole-volume analysis, not merely single-slice inference. By comparison, nnUNet processed only 21 volumes per hour and Swin-UNet processed 15 volumes per hour, confirming their impracticality for high-throughput screening.

These results substantiate that HARM-YOLO successfully balances detection accuracy with computational efficiency. The marginal 5.8% speed reduction relative to YOLOv10 is clinically acceptable given the substantial 8.1% gain in mAP@0.5 and 7.3% gain in sensitivity, particularly for small nodules where false negatives carry serious diagnostic consequences. The maintained real-time performance combined with state-of-the-art accuracy positions HARM-YOLO as a practical solution for deployment in large-scale lung cancer screening programs.

## 5 Discussion

The experimental results demonstrate that HARM-YOLO achieves significant improvements in automated lung nodule detection through architectural innovations designed for medical CT imaging. This section synthesizes the key findings, compares them to the broader literature, acknowledges limitations, and outlines future research directions.

### 5.1 Principal findings

HARM-YOLO outperformed existing frameworks across multiple metrics and datasets. On LIDC-IDRI, it achieved 90.8% mAP@0.5, surpassing YOLOv5 by 3.0%, ELCT-YOLO by 2.7%, and Faster R-CNN by 6.6%. On LUNA16, HARM-YOLO reached 92.5% mAP@0.5, outperforming YOLOv5 by 3.9% and segmentation-based models (nnUNet, Swin-UNet) by 5.5% and 5.1%, respectively. These gains reflect fundamental model enhancements, improving the capture of subtle morphological patterns and suppressing background interference.

Three architectural innovations contributed to this performance. First, the C2f-MDR module’s use of multi-dimensional dilated convolutions (Equations 1–5) expanded receptive fields, enabling simultaneous encoding of fine nodular textures and broader anatomical context. Ablation studies (**Table 7**) showed a 2.7% mAP@0.5 improvement over the YOLOv10 baseline, with particular gains for nodules ≤ 6mm. Second, the DENeck architecture (Equations 6–11) decoupled high- and low-level semantic pathways before fusion, yielding a 1.2% mAP gain and improving cross-dataset generalization. Third, the Background Attention Module (Equation 19) reduced false positives by 23% relative to YOLOv10 while maintaining 92.7% sensitivity. Grad-CAM visualizations (**Figure 5**) confirmed that attention mechanisms focused on nodule regions rather than vessels or pleural interfaces, addressing previous detector failures.

The model showed robustness across clinically relevant subgroups. For small nodules (≤ 6mm), HARM-YOLO achieved 84.9% mAP@0.5, outperforming YOLOv5 by 3.4% and classical detectors by 6.6–7.8% (**Table 5**). FROC analysis on LUNA16 showed 89.5% sensitivity at 1 false positive per scan, exceeding YOLOv5 by 5.4%. Cross-dataset experiments (**Table 4**) confirmed generalization, with HARM-YOLO maintaining 87.3% precision when transferring from LIDC-IDRI to LUNA16, compared to 85.2% for YOLOv5 and 80.4% for nnUNet. Statistical significance testing showed reproducibility with *p<* 0.01 for all comparisons (**Table 8**).

### 5.2 Comparison with prior work

This study advances existing literature in three key areas. First, prior YOLO-based detectors (MSG-YOLO, ELCT-YOLO) enhanced multi-scale fusion but retained fixed receptive fields that limited sensitivity to morphological diversity. HARM-YOLO’s SRFEM and PRFEM modules provide complementary receptive field expansion, capturing hierarchical patterns and multi-resolution features simultaneously. Ablation studies showed a 4.8% mAP gain, demonstrating that receptive field engineering yields more substantial improvements than scaling alone.

Second, comparison with segmentation-based methods highlights the trade-off between detection and segmentation. nnUNet and Swin-UNet had higher recall (1.2–1.5%) but lower precision (6.5–7.3%) compared to HARM-YOLO (**Tables 2**, **3**). HARM-YOLO’s one-to-one assignment strategy and background attention mechanism optimized detection precision, achieving superior F1-scores (0.918 *vs*. 0.855–0.863). Moreover, HARM-YOLO’s inference speed (2.7 seconds per scan) supports real-time integration, whereas segmentation models (7.2–8.7 seconds) are suited for offline batch processing.

Third, interpretability analyses distinguish HARM-YOLO from opaque deep learning systems. Grad-CAM and Score-CAM visualizations (**Figure 5**) showed that attention mechanisms focused on nodule regions, with a 0.78 mean IoU between attention heatmaps and ground truth, indicating strong alignment with clinical standards.

### 5.3 Limitations

Despite improvements, several limitations remain. First, evaluation was limited to LIDC-IDRI and LUNA16, which may not fully represent global population diversity in terms of scanner types and acquisition protocols. Multi-center validation across diverse cohorts is needed for broader generalizability, particularly in low- and middle-income countries. Cross-dataset experiments showed a 4.1% performance drop, highlighting sensitivity to domain shift.

Second, the current framework addresses detection but not downstream clinical tasks like malignancy risk scoring or Lung RADS categorization. Incorporating these features requires additional classification branches and training on richer datasets, such as NLST or UK Biobank. Without this integration, HARM-YOLO is limited to triage rather than comprehensive diagnostics.

Third, although computational efficiency is improved (3.2× faster than segmentation models), deployment on resource constrained hardware in community hospitals may not preserve real-time capability. Model compression and hardware-aware optimization techniques are needed to ensure accessibility across clinical settings.

Fourth, the model’s sensitivity to annotation quality and inter-observer variability requires further investigation. LIDC IDRI’s consensus ground truth may obscure meaningful uncertainty in small or ambiguous lesions. Probabilistic labeling or multi-annotator learning frameworks could improve robustness and provide calibrated confidence estimates.

### 5.4 Future directions and limitations

While HARM-YOLO achieves strong performance on 2D slice-based detection, several limitations and promising research directions merit discussion. The current framework’s reliance on axial 2D slices fundamentally constrains its ability to leverage volumetric spatial context that has been demonstrated to improve nodule characterization. Although 2D processing enables computational efficiency and compatibility with radiological workflows, it sacrifices inter-slice connectivity and the complete morphological envelope of nodules extending across multiple slices. Small nodules (≤ 6 mm) and juxtapleural lesions are particularly affected, as their morphological features often extend substantially in the z-axis. Future work should systematically investigate the performance trade-offs of 2.5D architectures that stack multiple adjacent slices as multi-channel inputs, offering a middle ground between computational feasibility and volumetric context utilization. Beyond geometric extensions, integrating temporal analysis across longitudinal screening exams represents a complementary direction. Automated growth assessment between serial scans enables detection of interval changes—a critical indicator of malignancy—that cannot be assessed from single time points. Incorporating temporal transformers or Siamese architectures for serial scan comparison could automate growth quantification and reduce false positive recalls, thereby enhancing malignancy risk stratification and screening efficiency.

Extending the framework to multi-center deployment requires addressing dataset limitations and domain shifts through federated learning. Recent federated learning algorithms with differential privacy guarantees offer promise for collaborative model training across institutions without centralizing sensitive patient data, addressing both privacy concerns and data heterogeneity inherent in real-world clinical settings. Simultaneously, enhancing clinical interpretability and trust demands integration of explainable AI techniques. Counterfactual explanations and concept-based visualization methods should be incorporated to provide radiologists with concrete evidence of decision boundaries and to explicitly align model activations with clinically meaningful features such as nodule spiculation, texture, and attachment patterns. This interpretability layer is essential for clinical acceptance and regulatory approval.

Finally, prospective clinical validation through reader studies is indispensable for establishing real-world utility. Comparative studies examining radiologist performance with and without HARM-YOLO assistance, following rigorous STARD guidelines and appropriate statistical power calculations, would provide definitive evidence of clinical value and inform implementation strategies for large-scale screening programs. Such trials should evaluate not only detection accuracy but also radiologist workflow efficiency, diagnostic confidence, and patient outcomes in diverse clinical settings.

## Data Availability

The original contributions presented in the study are included in the article/supplementary material. Further inquiries can be directed to the corresponding author.
